# Can reminder emails compel Americans to save? A two-million-person megastudy

**DOI:** 10.1093/pnasnexus/pgaf280

**Published:** 2025-09-01

**Authors:** Katherine L Milkman, Sean F Ellis, Dena M Gromet, Isabella M DeMay, Heather N Graci, Youngwoo Jung, Rayyan S Mobarak, Ramon A Silvera Zumaran, Mia Simmons-Yi, Christophe Van den Bulte, Shlomo Benartzi, Matthew Hilchey, Laura Goodyear, Dean Karlan, Nina Mazar, Daniel Mochon, Avni M Shah, Dilip Soman, Jonathan Zinman, Angela L Duckworth

**Affiliations:** Department of Operations, Information and Decisions, The Wharton School, University of Pennsylvania, Philadelphia, PA 19104, USA; Behavior Change for Good Initiative, The Wharton School and the School of Arts and Sciences, University of Pennsylvania, Philadelphia, PA 19104, USA; Behavior Change for Good Initiative, The Wharton School and the School of Arts and Sciences, University of Pennsylvania, Philadelphia, PA 19104, USA; Behavior Change for Good Initiative, The Wharton School and the School of Arts and Sciences, University of Pennsylvania, Philadelphia, PA 19104, USA; Behavioral Scientist, Washington, DC 20005, USA; Behavior Change for Good Initiative, The Wharton School and the School of Arts and Sciences, University of Pennsylvania, Philadelphia, PA 19104, USA; Department of Agricultural and Resource Economics, University of Maryland, College Park, MD 20742, USA; Department of Social and Decision Sciences, Carnegie Mellon University, Pittsburgh, PA 15213, USA; Mendoza College of Business, University of Notre Dame, South Bend, IN 46556, USA; Department of Marketing, The Wharton School, University of Pennsylvania, Philadelphia, PA 19104, USA; Anderson School of Management, University of California, Los Angeles, CA 90095, USA; Behavioural Economics in Action at Rotman, Rotman School of Management, University of Toronto, Toronto, ON, Canada M5S 3E6; Behavioural Economics in Action at Rotman, Rotman School of Management, University of Toronto, Toronto, ON, Canada M5S 3E6; Kellogg School of Management, Northwestern University, Evanston, IL 60208, USA; Questrom School of Business, Boston University, Boston, MA 02215, USA; A.B. Freeman School of Business, Tulane University, New Orleans, LA 70118, USA; Rotman School of Management, University of Toronto, Toronto, ON, Canada M5S 3E6; Department of Management, University of Toronto Scarborough, Toronto, ON, Canada M1C 1A4; Rotman School of Management, University of Toronto, Toronto, ON, Canada M5S 3E6; Department of Economics, Dartmouth College, Dartmouth, Hanover, NH 03755, USA; Department of Operations, Information and Decisions, The Wharton School, University of Pennsylvania, Philadelphia, PA 19104, USA; Department of Psychology, University of Pennsylvania, Philadelphia, PA 19104, USA

**Keywords:** savings, one-time transfer, recurring transfer, field experiment, megastudy

## Abstract

In the United States, 24% of adults have no savings and 39% have less than a month of income saved. We present results from a megastudy where nearly 2 million customers of a US bank were randomly assigned to receive one of seven different 2-month email campaigns, each employing a different behavioral science insight to nudge one-time and recurring savings deposits and increase savings balances or to a control condition without such messages. These campaigns increased the probability of making a one-time savings deposit, on average, by 0.05 percentage points (a 0.51% increase over control). The best-performing campaign delivered weekly messages to customers that differed depending on recent savings behavior: messages to customers who had not made a savings account deposit in the last week included a simple reminder to save, while those to customers who had made a savings account deposit in the prior week were congratulated on this accomplishment. This top-performing campaign increased the monthly likelihood that a customer made a one-time savings deposit by 0.13 percentage points (a 1.32% increase). We estimate that rolling this 2-month campaign out to everyone in our megastudy population would have led to an extra $6,123,996 to $9,910,090 in savings. Together, our findings highlight that light-touch, frequent email nudges can cost-effectively create small increases in savings deposits in the United States. Ideally, to generate meaningful benefits, behavioral science insights would be incorporated into a wider range of communications and incentives designed by financial institutions.

Significance Statement In the United States, increasing savings is a common goal. We present a 1,925,785-person megastudy testing seven different email campaigns encouraging one-time and recurring savings deposits to increase savings balances. Our campaigns increased the monthly probability of a one-time savings deposit, on average, by 0.05 percentage points (a 0.51% increase over our control condition). The best-performing campaign sent weekly savings reminders to people who had not saved in the last week and congratulatory messages to those who had successfully saved. This increased the monthly likelihood a customer made a one-time savings deposit by 1.32%. Deploying this campaign to everyone studied would have produced an estimated $6,123,996 to $9,910,090 in extra savings.

## Introduction

In the United States, 24% of adults have no savings whatsoever and 39% have less than a month of income saved (1). As a consequence, millions of Americans would be unable to cover emergency costs without selling a possession or taking on expensive debt (2, 3). Increasing savings is an urgent national priority, not only to ensure that Americans can cover unexpected expenses, but also because financial security confers numerous benefits, including improved health outcomes (4 ), greater happiness (5), and boosts in future income (6).

Past research suggests that insights from behavioral science can be leveraged to increase savings in low- and middle-income countries (7, 8) as well as in high-income countries (9–13). For example, because people tend to passively accept defaults, automatically enrolling employees in retirement savings programs and auto-escalating their contributions can increase savings (11). And likely because people find bite-sized goals more approachable, encouraging people to save $5 a day leads to substantially more savings than encouraging them to save $150 per month, despite the financial equivalence of these activities (9).

To the extent that savings failures are, in part, driven by action-intention gaps whereby people mean to set aside more each month but fail to follow through due to memory failures (14), reminders have the potential to increase savings. Past research has shown that in low-income countries (specifically, Bolivia, Peru, and the Philippines), reminding individuals of past commitments to save via text message (in Bolivia and the Philippines) or by mail (in Peru) increases their likelihood of meeting savings goals by an average of 5.4% (7). However, research is lacking in the United States on the efficacy of both savings reminders in general and theoretically distinct variations thereof. We examine whether a variety of different behaviorally informed email reminders can increase savings in the United States.^a^

We partnered with a large US bank to conduct a megastudy (19), testing the efficacy of seven different 2-month email campaigns (“interventions”) as a means of encouraging more savings. Bank customers (*n* = 1,925,785) were randomly assigned (i) to receive one of seven different sets of emails, all of which encouraged making one-time or recurring transfers to any of their savings accounts to boost their savings or, alternatively, (ii) to a business-as-usual control condition in which they received no savings reminders.

The goal of a megastudy is to provide an apples-to-apples comparison of the efficacy of different approaches to addressing a policy-relevant behavior challenge (19). This megastudy sought to address the challenge of increasing bank customers' savings contributions. Measuring the efficacy of behavioral interventions in the field—be they large or small—can help policymakers assess these interventions' cost-effectiveness and set appropriate expectations about what impact such interventions can achieve (20, 21). Although megastudies are poorly suited for testing theory or assessing the mechanism responsible for a given intervention's efficacy, they are well-suited for determining the most promising interventions for further testing and widespread deployment (22).

Here, different interventions were developed by separate teams of scientists with the goal of increasing monthly savings during a 2-month megastudy. Notably, scientists were restricted to scripting marketing email messages for the bank to send (which have notoriously low open rates (23)); scientists were not able to propose interventions via more attention-grabbing communication channels (e.g. website revisions, banners or pop-ups, in-app messaging, or text messages) or incentives for the bank to offer. In short, scientists were constrained to designing small information architecture interventions and were unable to change the bank's choice architecture.

## Megastudy methods

We conducted this megastudy in partnership with a large, well-respected international bank with over 3,000 branches and over 10,000 ATMs around the United States. The megastudy launched on 2022 March 1. The US bank customers were eligible for study inclusion if, as of 2022 February 28, they (i) were 18 or older, (ii) held checking and savings accounts with the bank that had been open for at least 90 days, (iii) had both 90-day average savings and checking account balances of more than $0 (and a savings account balance of no more than $25,000), (iv) had online access to their accounts, and (v) had opted into receiving marketing messages from the bank. From those who met these eligibility requirements, the bank randomly selected 1,999,164 customers to include in our megastudy.

**Fig. 1.**
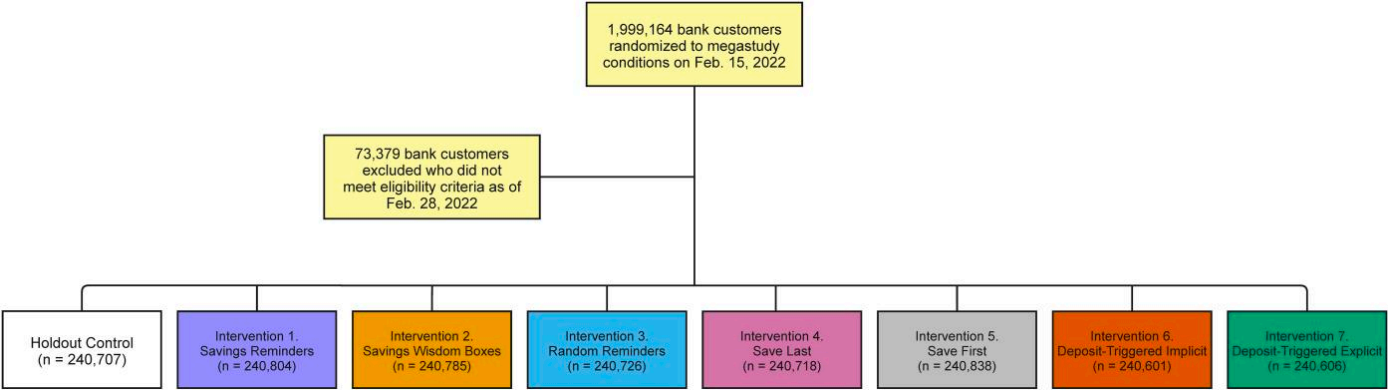
Megastudy CONSORT flow diagram.

Eligible bank customers were randomly assigned on 2022 February 15 to one of our megastudy's seven different treatment conditions or a business-as-usual control condition in which customers did not receive any of our treatment emails with reminders to save. The bank randomized customers to our eight different experimental conditions using the SAS command “proc surveyselect” without replacement. This command randomly selected 1/8 of the 1,999,164 customers eligible for randomization (249,896 customers) and assigned them to the first experimental condition. It then randomly selected another 249,896 customers out of the remaining 1,749,268 customers and assigned them to the second experimental condition. This process was repeated until all eight experimental conditions were assigned an equal share of customers. Because selection of participants and randomization to conditions occurred ∼2 weeks prior to sending the first emails (to allow time for email campaign configuration), 73,379 customers who were randomized to conditions did not meet at least one of our preregistered inclusion criteria on 2022 February 28 and were therefore excluded from our analytic sample, leaving us with a total of 1,925,785 customers in our megastudy (see Fig. 1 for CONSORT diagram). The average study condition included 240,723 customers (min = 240,601 customers, max = 240,838 customers).

To select the seven interventions launched in this megastudy, we invited 20 scientists and their collaborators to propose theoretically motivated approaches to increasing savings. From a total of 44 submitted intervention ideas, the principal investigators on this megastudy selected 17 interventions that they proposed to the bank for testing. The bank then ruled 10 of these infeasible, and a total of seven different interventions were ultimately deployed, testing a variety of theories outlined in Table 1 (column 3: “Theoretical rationale”).

**Table 1.** Description and theoretical rationale for each intervention condition.

Intervention (abbreviated name)	Description of email messaging campaign	Theoretical rationale
Intervention 1: weekly savings reminders (savings reminders)	Customers were sent an initial email explaining that they could expect to receive savings reminders each week when they had not made a savings deposit in the prior 7 days and congratulatory emails each week when they had made a savings deposit. All reminder emails encouraged customers to save immediately by scheduling a one-time or recurring savings transfer.	High-frequency reminders have proven more effective than lower-frequency reminders at preventing forgetting and increasing follow-through on a wide range of valuable activities ranging from vaccination to loan repayment (17, 24). Congratulatory statements have also been shown to enhance motivation and reinforce positive behaviors (25). Inviting people to save in order to earn congratulatory statements was expected to enhance motivation by providing a symbolic reward for saving (26).
Intervention 2: weekly savings reminders with wisdom boxes (savings wisdom boxes)	Customers were sent an initial email explaining that they could expect to receive savings reminders each week when they had not made a savings deposit in the prior 7 days and congratulatory emails containing a “Savings Wisdom Box” each week when they had made a savings deposit. Savings Wisdom Boxes were animated boxes that opened to reveal a “pearl of wisdom” about savings (e.g. “When it comes to saving money, endurance and persistence will be rewarded.”). All reminder emails encouraged customers to save immediately by scheduling a one-time or recurring savings transfer.	High-frequency reminders have proven more effective than lower-frequency reminders at preventing forgetting and increasing follow-through on a wide range of valuable activities ranging from vaccination to loan repayment (17, 24). “Wisdom boxes” containing “pearls of wisdom” were designed to delight and invoke curiosity (27) and to serve as symbolic rewards (26). Inviting people to save in order to earn a wisdom box was expected to enhance motivation over and above a congratulatory statement by providing a more curiosity-invoking and valued symbolic reward for saving (26).
Intervention 3: monthly random savings reminders (random reminders)	Customers were sent an email on one of 4 randomly selected dates in each month encouraging them to save immediately by scheduling a one-time or recurring savings transfer.	This condition provided reminders on random dates and was designed as a point of comparison for interventions that provided reminders following deposits (interventions 6 and 7).
Intervention 4: monthly reminders to save last (save last)	Customers were sent an email 5 days before the end of each month encouraging them to save what they had not yet spent by immediately scheduling a one-time or recurring savings transfer.	This condition provided reminders close to the end of the month because many people budget monthly (28, 29). Mental accounting suggests labels can change savings decisions (30, 31), and the message attempted to label “leftover” unspent funds at the end of a saving cycle as appropriate for savings.
Intervention 5: monthly reminders to save first (save first)	Customers were sent an email on the first day of each month encouraging them to save before spending by immediately by scheduling a one-time or recurring savings transfer.	This condition provided reminders close to the end of the month because many people budget monthly (28, 29). Mental accounting suggests labels can change savings decisions (30, 31), and the message attempted to label and cordon off a subset of the whole month's assets as “not yet spent” funds appropriate to set aside for savings.
Intervention 6: monthly implicit deposit-triggered savings reminders (deposit-triggered implicit)	Customers were sent an email encouraging them to save immediately by scheduling a one-time or recurring savings transfer the day after their checking account received its first deposit of $300 or greater that month.	People are more likely to save after receiving “windfalls,” or a significant influx of funds (32, 33). Capitalizing on this tendency and sending timely reminders to customers to save when they had just received a deposit in their checking account was intended to increase savings rates.
Intervention 7: monthly explicit deposit-triggered savings reminders (deposit-triggered explicit)	Customers were sent an email encouraging them to save from a recent deposit by scheduling a one-time or recurring savings transfer the day after their checking account received its first deposit of $300 or greater that month. The email stated: “a deposit was recently made to your account—save now.”	People are more likely to save after receiving “windfalls,” or a significant influx of funds (32, 33). Capitalizing on this tendency and sending timely reminders to customers to save when they had just received a deposit in their checking account was intended to increase savings rates. Unlike intervention 6, this intervention explicitly acknowledged a customer's recent windfall as a reason to save, providing a mental accounting justification for saving (30, 31).

Please refer to Figs. S1–S7 for the exact emails sent to customers in each intervention condition.

**Fig. 2.**
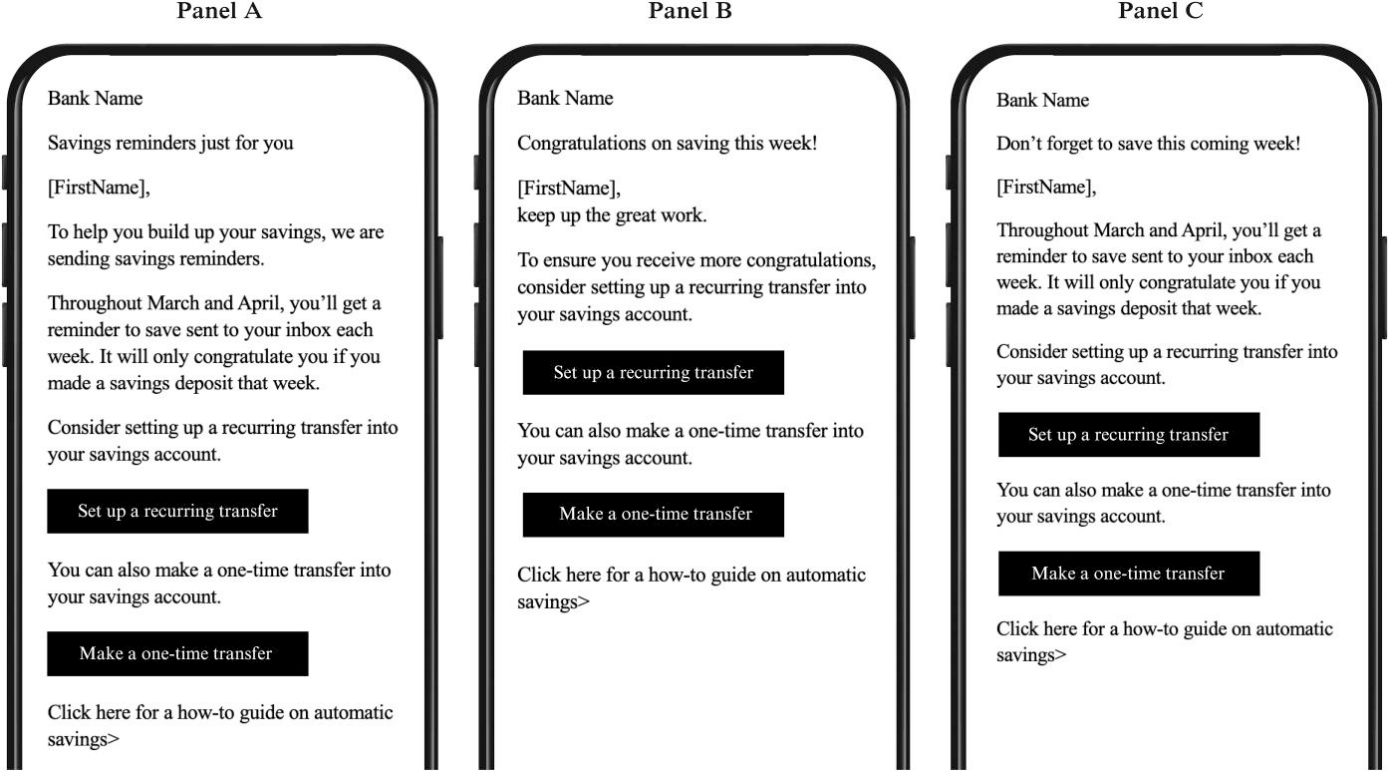
Illustration of the messages sent in intervention 1: weekly savings reminders email campaign. Panel A shows a stripped-down version of the initial email customers received setting their expectations for the messaging campaign, panel B shows a stripped-down version of the congratulatory email customers received on a given Friday if they had made a savings deposit in the week prior, and panel C shows a stripped-down version of the savings reminder email customers received on a given Friday if they had not made a deposit in the prior week. All graphics and formatting except for buttons customers were encouraged to click have been removed at the request of our bank partner to preserve their confidentiality.

The megastudy's seven treatment conditions test separate hypotheses about what works to encourage more saving. All conditions are described in detail in Tables 1 and 2, an illustrative email campaign is depicted in Fig. 2, and email stimuli from all campaigns are presented in Figs. S1–S7. All treatment conditions' emails promoted the transfer of money from checking to savings accounts and provided two buttons in emails for customers to click to “Set up a recurring transfer” or “Make a one-time transfer.” However, treatment conditions varied in content, time of delivery, the decision rules determining which emails would be sent when, and frequency of delivery (as detailed in Table 2).

**Table 2.** Description of each intervention condition's implementation.

Intervention (abbreviated name)	When were emails sent?	Number of emails sent to each customer per month (intended vs. observed)	Email trigger(s)	Email tailoring rules
Intervention 1: weekly savings reminders (savings reminders)	Initial email sent on 2022 March 1 Weekly emails sent every Friday thereafter	Intended: 5 Observed: 4.66	Fridays	Messages differed depending on whether the customer had made any deposit(s) into their savings accounts in the prior week
Intervention 2: weekly savings reminders with wisdom boxes (savings wisdom boxes)	Initial email sent on 2022 March 1 Weekly emails sent every Friday thereafter	Intended: 5 Observed: 4.66	Fridays	Messages differed depending on whether the customer had made any deposit(s) into their savings accounts in the prior week
Intervention 3: monthly random savings reminders (random reminders)	Initial email sent on one of 4 randomly selected dates in March 2022 Second email sent on one of 4 randomly selected dates in April 2022	Intended: 1 Observed: 0.99	“Random” dates: 2022 March 1 and 2022 April 5; 2022 March 4 and 2022 April 1; 2022 March 8 and 2022 April 12; or 2022 March 11 and 2022 April 8 (date pairs randomly selected for each customer)	None
Intervention 4: monthly reminders to save last (save last)	Initial email sent on 2022 March 25 Second email sent on 2022 April 26	Intended: 1 Observed: 0.98	Dates near the end of each month: 2022 March 25 and 2022 April 26	None
Intervention 5: monthly reminders to save first (save first)	Initial email sent on 2022 March 1 Second email sent on 2022 April 1	Intended: 1 Observed: 0.99	Dates at the start of each month: 2022 March 1 and 2022 April 1	None
Intervention 6: monthly implicit deposit-triggered savings reminders (deposit-triggered implicit)	One email sent the day after a customer's first ≥$300 deposit that month	Intended: 0.91 Observed: 0.56	First deposit of ≥$300 in a month	None
Intervention 7: monthly explicit deposit-triggered savings reminders (deposit-triggered explicit)	One email sent the day after a customer's first ≥$300 deposit that month	Intended: 0.91 Observed: 0.56	First deposit of ≥$300 in a month	None

Please refer to Figs. S1–S7 for the exact emails sent to customers in each intervention condition. The intended number of emails refers to the number of monthly emails called for by each email campaign we designed, while the observed number of emails reports the number that were actually sent (as explained in Methods), due to guidelines enforced by the bank's email system preventing customers from receiving a burdensome number of email messages within a 48-h period, some emails in our study were suppressed (e.g. because a customer had taken actions on a day with a scheduled email, like making several deposits, that triggered other, higher-priority bank emails).

Intervention 1: weekly savings reminders (or “savings reminders” for short) promised that any week a savings deposit was made, a congratulatory email message would arrive on Friday; in this treatment, those who did not make a deposit simply received Friday reminders to save. Intervention 2: weekly savings reminders with wisdom boxes (“savings wisdom boxes” for short) promised that deposits each week would result in a Friday email containing a “pearl of wisdom” (i.e. a brief, inspirational quote) that would emerge from an animated box (e.g. “when it comes to saving money, endurance and persistence will be rewarded”); in this treatment, as in the first, those who did not make a deposit simply received Friday reminders to save. Intervention 3: monthly random savings reminders (“random reminders” for short) sent emails encouraging savings on one of 4 randomly selected days each month, while “Intervention 4: monthly reminders to save last” (“save last” for short) encouraged people to save at the end of each month, setting aside any unspent money. Intervention 5: monthly reminders to save first (“save first” for short) tested the benefit of encouraging people to save at the beginning of each month, before incurring expenses. Interventions 6 and 7: monthly implicit deposit-triggered savings reminders (“deposit-triggered implicit” for short) and monthly explicit deposit-triggered savings reminders (“deposit-triggered explicit” for short) delivered emails that were triggered only when a customer made a checking account deposit of $300 or more. Deposit-triggered implicit (intervention 6) simply reminded the customer to save after such deposits without explicitly referencing the deposit, while deposit-triggered explicit (intervention 7) explicitly referenced the deposit as an opportunity to save. Table 1 details the theoretical rationale for each intervention (see column 3: “Theoretical rationale”).

The first email messages were sent at 9:05 AM ET on 2022 March 1, and the last messages were sent at 4:17 AM ET on 2022 April 29. Due to a limit on the number of e-mail messages bank customers can receive within a 48-h period to prevent excess messaging, 24.5% of customers did not receive every planned email in their experimental condition (e.g. because actions they had taken on a given day with a scheduled email, like making several deposits, triggered other, higher-priority bank emails). In addition, there were bugs in the deployment of two interventions that led some customers to miss planned emails, to receive them late, or to receive an email when they should not have.^b^ Following our preregistration, all of our analyses are conducted on an intent-to-treat basis, ignoring unsent or duplicate messages within any given experimental condition.

This megastudy was preregistered on Open Science Framework (OSF: https://bit.ly/3ATzK5D). Further, this research was approved by the University of Pennsylvania's Institutional Review Board, which granted a waiver of informed consent for the work. No personally identifying information about megastudy participants was shared with the researchers.

## Megastudy results

Bank customers in our megastudy were an average of 40.87 years old (SD = 14.95). No other demographic data on customers was shared to protect customer privacy. As of 2022 March 1, participants in our megastudy had been customers at the bank for an average of 10.22 years (SD = 8.51), held an average of $2,911.78 (SD = 5,559.34) in any of their savings accounts with the bank and held an average of $3,199.84 (SD = 13,210.78) in their checking accounts with the bank. As of 2022 February 28, our eight study conditions were balanced on the following metrics: customers' age, months since becoming a bank customer, use of online banking in the prior 90 days, possession of a credit card with the bank, whether they had made a one-time transfer into any of their savings accounts in the prior month, and average total checking and savings account balances with the bank in the prior 90 days (see Table 3 for details). However, we found a slight imbalance in whether participants had made a recurring transfer into any of their savings accounts in the prior month (see Table 3, *F*-test *P* = 0.032).

**Table 3.** Customer summary statistics and balance test results.

		Balance check variables								
	Customers	Credit card with the bank	Accessed online banking in the prior 90-days	Made at least one one-time transfer in the prior month	Made at least one recurring transfer in the prior month	Months with the bank	90-Day average balance of checking and savings accounts	90-Day average balance of checking accounts	90-Day average balance of savings accounts	Age
Total sample	1,925,785	47.39%	92.30%	9.94%	15.72%	122.68	$5,684.38	$2,945.23	$2,739.15	40.87
						(102.13)	(12,715.35)	(11,689.11)	(4,410.72)	(14.95)
Control condition	240,707	47.34%	92.32%	9.94%	15.63%	122.53	$5,691.59	$2,952.04	$2,739.55	40.86
						(102.07)	(13,695.66)	(12,739.78)	(4,419.16)	(14.95)
Intervention 1: savings reminders	240,804	47.48%	92.28%	9.99%	15.74%	123.05	$5,693.00	$2,944.78	$2,748.22	40.87
						(102.59)	(11,768.51)	(10,637.20)	(4,424.64)	(14.97)
Intervention 2: savings wisdom boxes	240,785	47.47%	92.37%	10.02%	15.86%	122.80	$5,703.13	$2,965.44	$2,737.69	40.88
						(102.05)	(13,035.99)	(12,042.63)	(4,399.06)	(14.94)
Intervention 3: random reminders	240,726	47.56%	92.26%	10.05%	15.69%	122.57	$5,666.56	$2,939.99	$2,726.57	40.87
						(101.95)	(12,040.95)	(10,960.14)	(4,398.34)	(14.93)
Intervention 4: save last	240,718	47.35%	92.34%	9.88%	15.52%	122.71	$5,685.60	$2,945.40	$2,740.20	40.88
						(102.10)	(11,820.76)	(10,705.50)	(4,420.71)	(14.96)
Intervention 5: save first	240,838	47.16%	92.28%	9.88%	15.75%	122.21	$5,670.53	$2,928.38	$2,742.15	40.85
						(101.56)	(13,410.24)	(12,441.08)	(4,411.76)	(14.96)
Intervention 6: deposit-triggered implicit	240,601	47.29%	92.27%	9.82%	15.84%	123.07	$5,677.41	$2,940.27	$2,737.14	40.90
						(102.91)	(13,153.83)	(12,168.46)	(4,401.36)	(14.95)
Intervention 7: deposit-triggered explicit	240, 606	47.47%	92.30%	9.91%	15.70%	122.52	$5,687.22	$2,945.55	$2,741.67	40.87
						(101.84)	(12,643.93)	(11,620.20)	(4,410.69)	(14.93)
*F*-statistic for *F*-test of equality across conditions		1.583	0.515	1.654	2.192	1.926	0.223	0.183	0.466	0.233
		*P* = 0.135	*P* = 0.824	*P* = 0.115	*P* = 0.032	*P* = 0.061	*P* = 0.980	*P* = 0.989	*P* = 0.860	P = 0.977

This table reports means for customer-level variables provided by our bank partner (and SDs in parentheses for continuous variables) for our full customer sample and for the subset of customers in each study condition as of 2022 February 28. Statistical tests involving multiple regression coefficients are all undirected.

Following our preregistration, our panel data analyses assess the monthly effect of our treatments on our primary outcome measure, which was the monthly change in customers' total savings account balances.^c^ Our secondary outcome measures, also analyzed month-by-month over time, were (i) a binary indicator for whether a customer made any one-time transfers into any of their savings accounts from another account at the bank during a given month and (ii) whether a customer made any recurring transfers into any of their savings accounts from another account at the bank during a given month (see SI Section 1: Definition of one-time and recurring transfers). All outcomes were calculated based on the bank's customer records.

Our dataset included monthly observations for each customer beginning a year prior to the start of our intervention period and continuing until the conclusion of our intervention period. As preregistered, we fit an ordinary least square (OLS) regression model to predict each outcome of interest over time. All panel data models controlled for customer fixed effects and month fixed effects. The key predictors in our regression were interactions between indicators for assignment to each of our megastudy's seven treatment conditions and an indicator for whether the month in question occurred during our study's 2-month intervention period (March to April 2022).^d^ SEs were clustered by customer.

We begin by describing the results of our interventions on our two most proximal preregistered secondary outcomes: one-time and recurring savings transfers. We then describe the results of our interventions on the more policy-relevant outcome of actual changes in savings, including our primary preregistered dependent measure.

### One-time savings transfers within the bank

We begin by analyzing the results of our treatments on customers' likelihood of making a one-time transfer into any of their savings accounts from another account at the bank during our study's 2-month intervention period. During this time, 9.88% of customers in the megastudy's business-as-usual control condition made a one-time transfer into any of their savings accounts from another account at the bank during a given month.

**Fig. 3.**
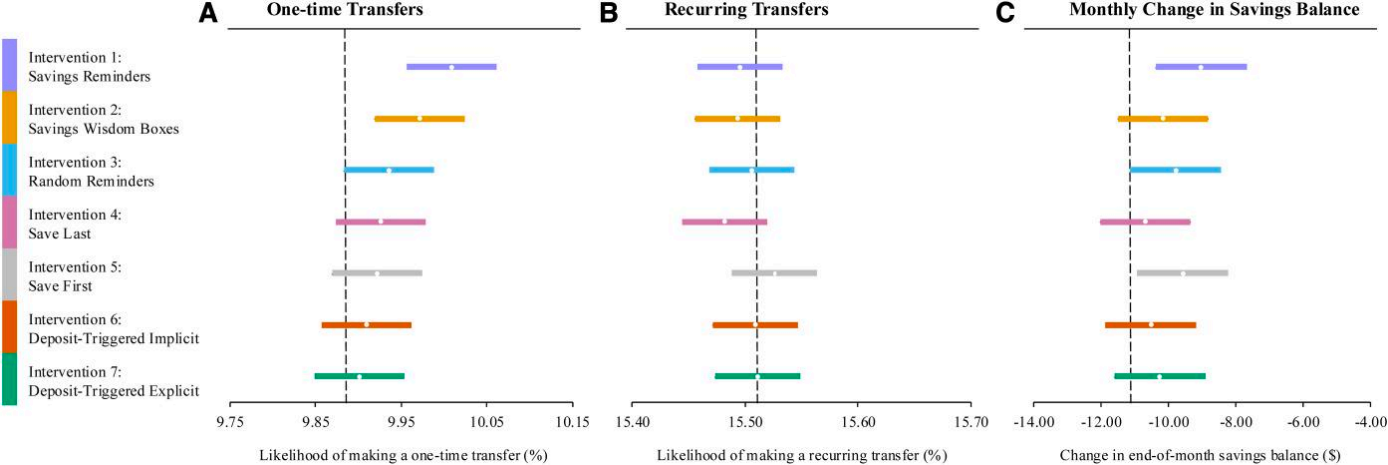
Average effects by intervention for each outcome. Dots indicate regression-estimated intervention effects with error bars depicting 95% CIs surrounding each estimate. Vertical dotted lines depict the business-as-usual control group average. Estimates are plotted for A) likelihood of making a one-time transfer to a savings account from another bank account in a given month, B) likelihood of making a recurring transfer to a savings account from another bank account in a given month, and C) monthly changes in cumulative savings account balance, with outliers winsorized at the 10th and 90th percentiles. Estimates are reported for (A) in Table 4, model 2, for (B) in Table 5, model 2, and for (C) in Table 6, model 6.

Figure 3 and Table 4 report the results of our preregistered regression models predicting monthly, one-time transfers into savings from other bank accounts during the 12 months prior to our study's launch and the 2 months of our megastudy (i.e. from 2021 March 1 through 2022 April 30). First, in an exploratory regression pooling all treatment conditions (Table 4, model 1), we find that reminding customers to save statistically significantly increases their likelihood of making a monthly, one-time transfer by 0.05 percentage points (95% CI = 0.016–0.092, *P* = 0.006), a 0.51% increase in transfers over the business-as-usual control condition. Turning to our preregistered analysis of the seven estimated treatment effects (Table 4, model 2), two treatments statistically significantly increased customers' monthly, one-time transfers after adjusting *P*-values computed using the Benjamini–Hochberg (BH) procedure, which controls for the false discovery rate when conducting multiple comparisons (34). Savings reminders (intervention 1), which entailed delivering a weekly reminder to save to those who had not made a savings deposit in the prior week or a message offering congratulations to those who had made a deposit in the prior week, statistically significantly increased the likelihood that a customer made a one-time savings deposit at some point in a month by 0.13 percentage points (95% CI = 0.073–0.177; a 1.32% increase). Savings wisdom boxes (intervention 2), which entailed delivering a promised weekly “pearl of wisdom” to those who had made a deposit in the prior week (or a reminder to save to those who had not), statistically significantly increased the likelihood that a customer made a one-time savings deposit at some point in a month by 0.09 percentage points (95% CI = 0.035–0.137; a 0.91% increase).^e^ Confidence sets for ranks (Table S1, model 1) showed that these are the only two treatments for which we cannot reject the null hypothesis (at 95% CI) that one of them is the true best-performing intervention. In a series of (post hoc) Wald tests not correcting for postselection inference, our treatment with the highest estimated treatment effect (intervention 1: savings reminders) outperforms five other treatments (BH-adjusted *P* ≤ 0.006) but does not statistically significantly outperform our savings wisdom boxes (intervention 2) treatment (Table S2). Further, we can reject the null hypothesis that all seven treatment effects are the same (*F* = 4.149, df = 6, *P* < 0.001). Notably, the email reminders sent at the highest frequency were the most effective at driving one-time savings transfers within the bank.

**Table 4.** Regression-estimated impact of our megastudy's seven intervention conditions on whether a customer made any one-time transfers into a savings account from another account at the bank during a given month of our 2-month intervention period, either pooling all intervention conditions (model 1), or by intervention condition (model 2).

	DV: made any one-time transfers to savings			
	Model 1		Model 2	
	*β*	*P*-value	*β*	BH-adjusted *P*-value
(Assigned any intervention) × (intervention period)	0.054**	0.006		
	(0.019)			
(Intervention 1: savings reminders) × (intervention period)			0.125***	<0.001
			(0.026)	
(Intervention 2: savings wisdom boxes) × (intervention period)			0.086**	0.003
			(0.026)	
(Intervention 3: random reminders) × (intervention period)			0.050	0.129
			(0.026)	
(Intervention 4: save last) × (intervention period)			0.040	0.211
			(0.026)	
(Intervention 5: save first) × (intervention period)			0.036	0.233
			(0.026)	
(Intervention 6: deposit-triggered implicit) × (intervention period)			0.024	0.418
			(0.026)	
(Intervention 7: deposit-triggered explicit) × (intervention period)			0.016	0.533
			(0.026)	
*F*-statistic for *F*-test of equality across seven study conditions			4.149	<0.001
Observations	26,960,990		26,960,990	
Customer fixed effects	1,925,785		1,925,785	
Month fixed effects	14		14	
*R* ^2^	0.910		0.910	

This table reports the results of two OLS regressions predicting whether a given customer made any one-time transfers into their savings account at the bank in a given month. Both models include 14 months of observations for each customer (from March 2021 to April 2022), and both models include customer fixed effects as well as month fixed effects. In model 1, the primary predictor variable is an interaction between an indicator for whether the observation month fell during the study's 2-month intervention period and an indicator for whether a customer was assigned to receive any of our megastudy's seven intervention conditions. In model 2, the primary predictor variables are interactions between an indicator for whether the observation month fell during the study's 2-month intervention period and separate indicators for whether a customer was assigned to each of our megastudy's seven intervention conditions. Regression coefficients and SEs have been multiplied by 100 to improve interpretability. SEs reported in parentheses are clustered at the customer level, and *P*-values in model 2 are adjusted for multiple comparisons using the BH procedure.

*P < 0.05.

^**^ *P* < 0.01.

^***^ *P* < 0.001.

**Table 5.** Regression-estimated impact of our megastudy's seven intervention conditions on whether a customer made any recurring transfers into a savings account from another account at the bank during a given month of our 2-month intervention period, either pooling all intervention conditions (model 1) or by intervention condition (model 2).

	DV: made any recurring transfers to savings			
	Model 1		Model 2	
	*β*	*P*-value	*β*	BH-adjusted *P*-value
(Assigned any intervention) × (intervention period)	−0.007	0.618		
	(0.014)			
(Intervention 1: savings reminders) × (intervention period)			−0.015	0.751
			(0.019)	
(Intervention 2: savings wisdom boxes) × (intervention period)			−0.017	0.751
			(0.019)	
(Intervention 3: random reminders) × (intervention period)			−0.004	0.969
			(0.019)	
(Intervention 4: save last) × (intervention period)			−0.028	0.751
			(0.019)	
(Intervention 5: save first) (intervention period)			0.016	0.751
			(0.019)	
(Intervention 6: deposit-triggered implicit) × (intervention period)			−0.002	0.969
			(0.019)	
(Intervention 7: deposit-triggered explicit) × (intervention period)			7.294E−04	0.969
			(0.019)	
*F*-statistic for *F*-test of equality across seven study conditions			1.146	0.332
Observations	26,960,990		26,960,990	
Customer fixed effects	1,925,785		1,925,785	
Month fixed effects	14		14	
*R* ^2^	0.955		0.955	

This table reports the results of two OLS regressions predicting whether a given customer made any recurring transfers into their savings account at the bank in a given month. Both models include 14 months of observations for each customer (from March 2021 to April 2022), and both models include customer fixed effects as well as month fixed effects. In model 1, the primary predictor variable is an interaction between an indicator for whether the observation month fell during the study's 2-month intervention period and an indicator for whether a customer was assigned to receive any of our megastudy's seven intervention conditions. In model 2, the primary predictor variables are interactions between an indicator for whether the observation month fell during the study's 2-month intervention period and separate indicators for whether a customer was assigned to each of our megastudy's seven intervention conditions. Regression coefficients and SEs have been multiplied by 100 to improve interpretability. SEs reported in parentheses are clustered at the customer level, and *P*-values in model 2 are adjusted for multiple comparisons using the BH procedure.

*P < 0.05.

**P < 0.01.

***P < 0.001.

**Table 6.** Regression-estimated impact of our megastudy's seven intervention conditions on the change in a customer's total savings balances during a given month of our 2-month intervention (i) winsorizing this outcome at the first and 99th percentiles (models 1 and 2), at the fifth and 95th percentiles (models 3 and 4), at the 10th and 90th percentiles (models 5 and 6) or (ii) reshaping this outcome using an IHS transformation; either pooling all intervention conditions (models 1, 3, 5, and 7) or by intervention condition (models 2, 4, 6, and 8).

	DV: Δ in monthly savings winsorized at the first and 99th pctls				DV: Δ in monthly savings winsorized at the fifth and 95th pctls				DV: Δ in monthly savings winsorized at the 10th and 90th pctls				DV: IHS (Δ in monthly savings)			
	Model 1		Model 2		Model 3		Model 4		Model 5		Model 6		Model 7		Model 8	
	*β*	*P*-value	*β*	BH-adjusted *P*-value	*β*	*P*-value	*β*	BH-adjusted *P*-value	*β*	*P*-value	*β*	BH-adjusted *P*-value	*β*	*P*-value	*β*	BH-adjusted *P*-value
(Assigned any intervention) × (intervention period)	1.691	0.419			1.898	0.075			1.250*	0.039			0.014	0.104		
	(2.091)				(1.065)				(0.606)				(0.009)			
(Intervention 1: savings reminders) × (intervention period)			3.852	0.672			3.596	0.076			2.326*	0.026			0.034 *	0.019
			(2.772)				(1.412)				(0.803)				(0.011)	
(Intervention 2: savings wisdom boxes) × (intervention period)			1.664	0.859			1.535	0.399			1.048	0.303			0.014	0.382
			(2.758)				(1.408)				(0.802)				(0.011)	
(Intervention 3: random reminders) × (intervention period)			3.603	0.672			2.803	0.162			1.558	0.121			0.017	0.382
			(2.761)				(1.408)				(0.802)				(0.011)	
(Intervention 4: save last) × (intervention period)			−0.283	0.941			0.401	0.776			0.432	0.590			4.924E−04	0.966
			(2.764)				(1.409)				(0.802)				(0.011)	
(Intervention 5: save first) × (intervention period)			0.203	0.941			2.127	0.304			1.684	0.121			0.016	0.382
			(2.762)				(1.407)				(0.801)				(0.011)	
(Intervention 6: deposit-triggered implicit) × (intervention period)			1.396	0.859			1.336	0.399			0.705	0.442			0.008	0.591
			(2.766)				(1.406)				(0.801)				(0.011)	
(Intervention 7: deposit-triggered explicit) × (intervention period)			1.398	0.859			1.486	0.399			0.993	0.303			0.009	0.591
			(2.767)				(1.410)				(0.803)				(0.011)	
*F*-statistic for *F*-test of equality across seven study conditions			0.637	0.701			1.107	0.356			1.301	0.253			1.709	0.114
Observations	24,124,139		24,124,139		24,124,139		24,124,139		24,124,139		24,124,139		24,124,139		24,124,139	
Customer fixed effects	1,925,785		1,925,785		1,925,785		1,925,785		1,925,785		1,925,785		1,925,785		1,925,785	
Month fixed effects	13		13		13		13		13		13		13		13	
*R* ^2^	0.068		0.068		0.101		0.101		0.120		0.120		0.145		0.145	

This table reports the results of eight OLS regressions predicting the change in a customer's total savings account balances during a given month after winsorization or IHS transformation. All models include 13 months of observations for each customer (from April 2021 to April 2022), and all models include customer fixed effects as well as month fixed effects. In models 1, 3, 5, and 7, the primary predictor variable is an interaction between an indicator for whether the observation month fell during the study's 2-month intervention period and an indicator for whether a customer was assigned to receive any of our megastudy's seven intervention conditions. In models 2, 4, 6, and 8, the primary predictor variables are interactions between an indicator for whether the observation month fell during the study's 2-month intervention period and separate indicators for whether a customer was assigned to each of our megastudy's seven intervention conditions. SEs reported in parentheses are clustered at the customer level, and *P*-values in models 2, 4, 6, and 8 are adjusted for multiple comparisons using the BH procedure.

^*^ *P* < 0.05.

**P < 0.01.

***P < 0.001.

### Recurring savings transfers within the bank

Next, we turn to analyzing the results of our treatments on customers' likelihood of making a recurring transfer into any of their savings accounts from another account at the bank during our 2-month intervention period. Like one-time savings transfers, recurring transfers were encouraged in all treatment emails, in this case with a button that said “Set up a recurring transfer” (Figs. S1–S7). Unfortunately, while it was possible for customers to set up one-time transfers in the bank's app, it was not possible for customers to set up a recurring transfer there, which is where customers were directed if they accessed emails from a mobile device and clicked the button encouraging them to “Set up a recurring transfer.”

Figure 3 and Table 5 report the results of our preregistered regression models predicting recurring transfers into savings from another account at the bank each month during the 12 months prior to our study's launch and in the 2 months of our megastudy (i.e. from 2021 March 1 to 2022 April 30). In the megastudy's business-as-usual control condition, 15.51% percent of customers made a recurring transfer into any of their savings accounts during a given month in our intervention period. In an exploratory regression pooling all treatment conditions (Table 5, model 1), we find that reminding people to save has no statistically significant effect on people's likelihood of making a recurring transfer to any of their savings accounts each month. Turning to our preregistered analysis of the seven estimated treatment effects (Table 5, model 2), we find that no individual treatment had a statistically significant effect on making recurring transfers either.

### Changes in monthly savings account balances

Finally, we analyze our primary, preregistered dependent variable, which is the most policy-relevant of our outcomes of interest and is downstream of the two events analyzed so far: the (winsorized) change in a customer's monthly savings balance. Prior to winsorization, this outcome was symmetrically distributed, but it had extreme kurtosis with very long tails (e.g. without adjustment, the minimum value of our outcome = −$1,069,208; first pctl = −$4,800; 99th pctl = $5,784; max value = $2,000,015; kurtosis = 29,842; see Fig. S8), which we anticipated in our preregistration. Accordingly, we repeat our standard OLS regression analysis with several preregistered data transformations to address outliers. Specifically, following our preregistration, we report regression models predicting our primary outcome variable: (i) winsorized at the 99th and first percentiles, (ii) winsorized at the 95th and fifth percentiles, (iii) winsorized at the 90th and 10th percentiles, and (iv) transformed by taking its inverse hyperbolic sine (IHS). See Fig. S8 for histograms of the change in customers' monthly savings balances during our intervention period, both unadjusted and following each of the 4 aforementioned preregistered transformations.^f^

Figure 3 and Table 6 report the results of our preregistered regression models predicting monthly changes in customers' savings balances (transformed in each of the 4 ways described above) during the 11 months prior to our study's launch and the 2 months of our megastudy (i.e. from 2021 April 1 to 2022 April 30).^g^

First, when we analyze changes in customers' monthly savings balances winsorizing at only the 99th and first percentiles (our primary preregistered transformation), we find no statistically significant effects of our treatments, either when we pool all treatment conditions together (Table 6, model 1) or when we analyze treatment conditions separately (Table 6, model 2). Winsorizing at the 95th and fifth percentiles yields a marginally statistically significant positive effect of our pooled treatment (*P* = 0.075, see Table 6, model 3) and a marginally statistically significant positive effect of the treatment with the largest regression-estimated effect—savings reminders (intervention 1), which delivered a weekly reminder to save to those who had not made a savings deposit in the prior week or, instead, a message offering congratulations to those who had (BH-adjusted *P* = 0.076, see Table 6, model 4).

Winsorizing at the 90th and 10th percentiles and pooling all treatment conditions (Table 6, model 5), we find that reminders statistically significantly increase monthly savings balances by $1.25 (95% CI = 0.06–2.44, *P* = 0.039), on average—a 25.25% increase in savings growth over the business-as-usual control condition, where customers' monthly change in savings balances averaged −$4.95 (SD = 371.06). Turning to our seven estimated treatment effects (Table 6, model 6), we find that just one treatment statistically significantly increased savings: savings reminders (intervention 1) increased people's savings balances by $2.33, on average—a 47.07% increase in savings growth over the business-as-usual control condition (95% CI = 0.75–3.90, BH-adjusted *P* = 0.026). In a series of post hoc Wald tests not correcting for postselection inference, this treatment does not outperform any of the other treatment conditions (BH-adjusted *P* ≥ 0.110) after adjusting for multiple comparisons (Table S3). Further, we cannot reject the null hypothesis that all seven estimated treatment effects are the same (*F* = 1.301, df = 6, and *P* = 0.253). Finally, confidence sets for ranks (Table S1, model 4) show that we can reject the null hypothesis (at 95% CI) that a treatment is the true best only for the treatment with the lowest measured effect (intervention 7: deposit-triggered explicit). Any of the other six could be the true best.

Finally, applying an IHS transformation shows no statistically significant effect of our pooled treatment (*P* = 0.104, see Table 6, model 7) but a statistically significant positive effect of the treatment with the largest regression-estimated effect (BH-adjusted *P* = 0.019, see Table 6, model 8).

To better understand the source of the small changes in savings detected in some winsorized analyses, we ran additional exploratory analyses that were not preregistered, repeating our primary regression analysis (from Table 4, model 2) with three alternative dependent variables: (i) the total number of one-time transfers to savings a customer made during a given month, (ii) the average size of one-time savings transfers during a given month, and (iii) the maximum size of any savings transfer made during a given month. As depicted in Table S4, these analyses show that weekly savings reminders (intervention 1) produce a significant, 0.003 percentage point increase in the total number of one-time transfers a customer makes in a given month during the intervention period (see model 2, 95% CI = 0.001–0.004, BH-adjusted *P* < 0.001). However, this intervention does not produce any significant changes in the average (see model 4, 95% CI = −0.964–1.282, BH-adjusted *P* = 0.932) or maximum size (see model 6, 95% CI = −0.977–1.389, BH-adjusted *P* = 0.877) of transfers to savings. These results suggest that any increase in monthly savings produced by weekly savings reminders (intervention 1) is being driven by increases in the presence of one-time transfers to savings, but not increases in the size of these transfers to savings.

### Analysis of heterogeneity of treatment effects

We examine whether any of the estimated treatment effects from our preregistered analyses differ significantly as a function of 37 different observable customer characteristics, including age, checking account balance preintervention, and income (see SI Section 2: Heterogeneity analyses). After correcting for multiple hypothesis testing using both the BH procedure and the Benjamini–Yekutieli (BY) procedure (35), no results reach statistical significance at the 5% level (all BY-adjusted *P* ≥ 0.718).

### Analysis of postintervention treatment effects

We also examine whether any of the aforementioned treatment effects endure in the 1, 2, and 3 months after the conclusion of our 2-month intervention period. To do this, we employ the same regression framework as in our primary analyses but add (i) separate indicators to our models for observations from 1 month postintervention, 2 months postintervention, and 3 months postintervention and (ii) interactions between each of these three new indicators and all treatment indicators (see SI Section 3: Post-intervention analyses).

We find that reminders (when pooled) produce an estimated 0.08 percentage point increase in the likelihood of any one-time transfers to savings in the 1 month postintervention (Table S5, model 1; 95% CI = 0.025–0.132, *P* = 0.004), or a 0.90% increase in the likelihood of such transfers over the business-as-usual control condition. This is not surprising given that (i) many customers likely read the savings reminder emails sent late in the intervention period during the month postintervention (since all emails are not read on the day they are sent) and (ii) many transfers scheduled after reading reminder emails sent late in the intervention period would take place shortly afterwards in the 1 month following the intervention period. However, 2 and 3 months postintervention, the estimated effects of reminders (pooled) on the likelihood of any one-time transfers drop below standard levels of statistical significance (both *P* > 0.05). Similarly, while several interventions produce significant increases in the likelihood of a customer making any one-time transfers to savings in the 1-month postintervention after correcting for multiple hypotheses testing (Table S5, model 2), no interventions produce significant increases in this outcome in the 2 or 3 months postintervention.

Turning to our other dependent variables, we do not measure any significant durable effects of our interventions either on customers' likelihood of making any recurring transfers (Table S6) or on their changes in monthly savings (Table S7).

## Discussion

The results from this megastudy suggest that even an extraordinarily light-touch, behaviorally informed intervention—a reminder email campaign—can effectively nudge consumers in high-income countries to make slightly more one-time savings transfers. We also find that such campaigns can produce small increases in savings in the United States in analyses that take a particularly aggressive approach to trimming outliers. While not predicted a priori, we note that the savings reminder messages that were most effective were sent on a weekly basis (whereas all other reminders were sent, at most, on a monthly basis). Specifically, the best-performing email campaign, savings reminders (intervention 1), delivered a weekly reminder to save to those who had not made a savings deposit in the prior week or, instead, a message offering congratulations to those who had. This campaign increased the monthly likelihood that a customer made a one-time savings deposit by 0.13 percentage points (a 1.32% increase) and—in a model winsorizing the top and bottom 10% of outliers—it increased monthly savings by $2.33 (a 47.07% increase).

We estimate that our intervention messages collectively led to an additional 181,988 one-time savings deposits during our 2-month intervention (95% CI: 53,922–310,054).^h^ Extrapolating from our estimates of the best-performing campaign's impact on savings in our preregistered models winsorizing the top and bottom 1, 5, and 10% of outliers after applying the James–Stein shrinkage procedure, we estimate that rolling this 2-month campaign out to everyone in our megastudy population would have led to an extra $6,123,996 to $9,910,090 in savings.^i^ Our findings highlight that light-touch, low-cost email-based reminder campaigns can encourage small increases in savings deposits in the United States.

This study has several important limitations. First, interventions were only delivered for a 2-month period; future research is needed to assess the durability of measured treatment effects over longer time periods. If effects endure during longer campaigns, then lengthy email campaigns could produce more meaningful benefits. Second, the savings outcomes measured captured just one form of liquid savings rather than overall wealth (though recent work suggests it is unlikely that the additional savings people were nudged to set aside were financed with expensive debt; 36). Still, future research capturing a broader set of financial outcomes would be valuable. Third, as noted earlier, the hyperlinks in our emails that were intended to help customers set up recurring transfers to any of their savings accounts in fact led smartphone users to a mobile app that did not allow recurring transfers, which likely limited the impact of our messages. Future research nudging recurring savings transfers with improved smartphone functionality would be valuable. Fourth, our findings may be context-dependent: we do not know how well they would generalize to other cultures, banking platforms, or new communication platforms that takeover as technology advances, which will make future replications in new contexts valuable. Finally, since megastudies are, by design, aimed at identifying the most promising approach(es) to promoting a given behavior but not necessarily unpacking mechanism, follow-up studies are needed to replicate these effects, assess their robustness, and examine their mechanisms (22). In particular, it would be valuable for future research to disentangle whether the efficacy of the top-performing messages in our campaign (intervention 1: savings reminders and intervention 2: savings wisdom boxes) was primarily driven by the frequency at which reminders were sent or the content of the messaging.

Because email messaging is nearly free (as the costs are primarily associated with fixed design and setup time; 37), our results suggest that sending Americans weekly email reminders to save would likely be a cost-effective way for financial institutions to slightly increase their customers' savings (20), at least over an interval of 2 months.^j^ And low-cost nudges with very small benefits can create meaningful value when deployed at scale (20, 24).

The potential benefits of behavioral insights to financial institutions seeking to boost Americans' savings rates are likely underestimated by this study. In past research, email has proven a less potent medium for communications designed to change behavior than direct mail, phone calls, or text messaging (39), likely due to low open rates and limited attention (23). Given that e-mail nudges nevertheless yielded measurable behavior change in this megastudy, our findings suggest it would be valuable for the US financial institutions to field test a wider range of behaviorally informed approaches to communicating with their customers about savings. More effective opportunities for leveraging behavioral insights to increase savings could likely be identified by, for example, allowing behavioral scientists to field test redesigned websites, incentive schemes, apps, text messages, direct mail, and other forms of communication. In other words, these results suggest that behavioral scientists likely need more sand in their sandbox to build more powerful interventions.

## Supplementary Material

pgaf280_Supplementary_Data

## Data Availability

The experimental data analyzed in this article were provided by our bank partner. The bank fully anonymized and de-identified all data before we were granted access to it. To protect customers' secure financial data and privacy, we cannot publicly post individual-level financial data. Analysis scripts have been deposited in the Open Science Framework (https://osf.io/zkgp8).

## Notes

a While customers in our study had not explicitly indicated that they wanted to boost their savings, the majority of Americans have the goal of saving more (15). We follow a large past literature studying messages to encourage behaviors that are widely viewed as goal-aligned (e.g. medication adherence and vaccination) and refer to our messages as “reminders” (16–18).

b During the treatment period, customers in “Intervention 5: deposit-triggered implicit” and “Intervention 6: deposit-triggered explicit” (Table 2) who received their emails late after making a deposit of ≥$300 typically received them 2 to 4 days after making a qualifying deposit, rather than at our intended 1-day delay.

c Following the Bank’s definition of savings accounts, this category includes several standard types of savings accounts (with a range of interest rates and fees) as well as money market accounts.

d An indicator for assignment to our business-as-usual control condition was omitted.

e We applied the James–Stein shrinkage procedure to account for the fact that the largest and second largest of seven treatment effects are likely to be upwardly biased (the winner’s curse). This procedure estimates that the two treatments with significant effects generated a 0.11 percentage point (1.11%) increase and 0.08 percentage point (0.81%) increase in one-time savings deposits, respectively.

f Note that we did not preregister analyzing this outcome unadjusted given our anticipation of extreme outliers.

g Note that we only obtained data on customers’ savings balances for 12 months prior to the start of our intervention, which means that when examining changes in savings balances, we can only calculate this difference variable for the 11 months preintervention and the two months of our intervention period.

h We calculate the number of additional one-time transfers induced by our seven interventions by multiplying the pooled treatment effect from Table 4, model 1 (0.054; 95% CI: 0.016–0.092) by the number of customers in our seven treatment conditions (*n* = 1,685,078), and we then multiply this number by two because our intervention lasted for two months and our treatment effect was estimated on a monthly basis.

i We estimate total increase in savings induced by our top-performing intervention by (i) applying the James–Stein shrinkage procedure to adjust our estimate of this intervention’s effect (yielding an estimated treatment effect of 1.590 when applied to Table 6, model 2, 2.573 when applied to Table 6, model 4, and 1.774 when applied to Table 6, model 6), (ii) multiplying this estimate by the total number of customers across all conditions in our megastudy (*n* = 1,925,785), and (iii) multiplying this number by two (because our intervention lasted for 2 months but its effect was estimated on a monthly basis).

j Of course, there may be welfare costs associated with adding to vying for consumers’ limited attention with additional reminders (see Allcott and Kessler (38) for a discussion). Further analysis of such attentional costs would be worthwhile.
