# Correction to: Can reminder emails compel Americans to save? A two-million-person megastudy

**DOI:** 10.1093/pnasnexus/pgag268

**Published:** 2026-08-18

This is a correction to: Katherine L Milkman, Sean F Ellis, Dena M Gromet, Isabella M DeMay, Heather N Graci, Youngwoo Jung, Rayyan S Mobarak, Ramon A Silvera Zumaran, Mia Simmons-Yi, Christophe Van den Bulte, Shlomo Benartzi, Matthew Hilchey, Laura Goodyear, Dean Karlan, Nina Mazar, Daniel Mochon, Avni M Shah, Dilip Soman, Jonathan Zinman, Angela L Duckworth, Can reminder emails compel Americans to save? A two-million-person megastudy, *PNAS Nexus*, Volume 4, Issue 9, September 2025, pgaf280, https://doi.org/10.1093/pnasnexus/pgaf280

A change has been made to the author list.

