# Supplementary material for: Can reminder emails compel Americans to save? A two-million-person megastudy
Source: PNAS Nexus. 2025 Sep 1;4(9):pgaf280. doi: 10.1093/pnasnexus/pgaf280 (PMC12477529; doi:10.1093/pnasnexus/pgaf280)
Supplement: pgaf280_Supplementary_Data [file pgaf280_supplementary_data.docx]

**TITLE:** Can reminder emails compel Americans to save? A two million person megastudy

**AUTHORS:** Katherine L. Milkman^1,*^, Sean F. Ellis^2^, Dena M. Gromet^2^, Isabella M. DeMay^2^, Heather N. Graci^3^, Youngwoo Jung^2^, Rayyan S. Mobarak^4^, Ramon A. Silvera Zumaran^5^, Mia N. Simmons^6^, Christophe Van den Bulte^7^, Shlomo Benartzi^8^, Matthew Hilchey^9^, Laura Goodyear^9^, Dean Karlan^10^, Nina Mazar^11^, Daniel Mochon^12^, Avni M. Shah^13,14^, Dilip Soman^13^, Jonathan Zinman^15^, Angela L. Duckworth^1,16^

**AFFILIATIONS:**

^1^ Department of Operations, Information and Decisions, The Wharton School, University of Pennsylvania, Philadelphia, PA 19104, USA

^2^ Behavior Change for Good Initiative, The Wharton School and the School of Arts & Sciences, University of Pennsylvania, Philadelphia, PA 19104, USA

^3^ Behavioral Scientist, Washington, DC 20005, USA

^4^ Department of Agricultural and Resource Economics, University of Maryland, College Park, MD 20742, USA

^5^ Department of Social and Decision Sciences, Carnegie Mellon University, Pittsburgh, PA 15213, USA

^6^ Mendoza College of Business, University of Notre Dame, South Bend, IN 46556, USA

^7^ Department of Marketing, The Wharton School, University of Pennsylvania, Philadelphia, PA 19104, USA

^8^ Anderson School of Management, University of California, Los Angeles, CA 90095, USA

^9^ Behavioural Economics in Action at Rotman, Rotman School of Management, University of Toronto, Toronto, ON M5S 3E6, Canada

^10^ Kellogg School of Management, Northwestern University, Evanston, IL 60208, USA

^11^ Questrom School of Business, Boston University, Boston, MA 02215, USA

^12^ A.B. Freeman School of Business, Tulane University, New Orleans, LA 70118, USA

^13^ Rotman School of Management, University of Toronto, Toronto, ON M5S 3E6, Canada

^14^ Department of Management, University of Toronto Scarborough, Toronto, ON M1C 1A4, Canada

^15^ Department of Economics, Dartmouth College, Dartmouth, Hanover, NH 03755, USA

^16^ Department of Psychology, University of Pennsylvania, Philadelphia, PA 19104, USA

*Corresponding author, email: kmilkman@wharton.upenn.edu.

**Table of Contents**

[1. Definition of one-time and recurring transfers 3](#_sd1y1lh2qw8c)

[2. Heterogeneity analyses 4](#_epnxpcezw0s2)

[3. Post-intervention analyses 6](#_iv5av77o7goz)

[4. Robustness check: Customers who did not have accounts in the pre-intervention period 6](#_o3kkbppotufw)

[5. Robustness check: Dropping customers with shared accounts 7](#_28lsijfaw964)

[6. Figures 9](#_qo519s33hhxy)

[Figure S1. Illustration of the emails customers received if they were assigned to Intervention 1 9](#_dk21udk03d8m)

[Figure S2. Illustration of the emails customers received if they were assigned to Intervention 2 10](#_3ncufkisms0d)

[Figure S3. Illustration of the emails customers received if they were assigned to Intervention 3 11](#_tw6jv6kfg6fv)

[Figure S4. Illustration of the emails customers received if they were assigned to Intervention 4 12](#_sty1jby6o1)

[Figure S5. Illustration of the emails customers received if they were assigned to Intervention 5 13](#_uy4wocn91moc)

[Figure S6. Illustration of the emails customers received if they were assigned to Intervention 6 14](#_mue6yll5er6)

[Figure S7. Illustration of the emails customers received if they were assigned to Intervention 7 15](#_t73y1qrjpv6f)

[Figure S8. Histograms depicting the distribution of changes in customers’ total savings balances per month during our two month intervention period and the eleven months prior. 16](#_g77ibvpibo5s)

[7. Tables 18](#_e4dh3xpw7dzf)

[Table S1. Test of ranks of interventions. 18](#_b13rj5k79fqj)

[Table S2.](#_me7ee8ybv2hv) [Wald Tests comparing Intervention 1 to other interventions targeting one-time transfers](https://docs.google.com/document/d/1xPrzD0ThBi105s64vfgLNnuWY6IJTvj5VoeUq2MCXDs/edit#heading=h.me7ee8ybv2hv) [19](#_me7ee8ybv2hv)

Table S3. Wald Tests comparing Intervention 1 to other interventions targeting changes in customers’ savings balances. [20](#_swnlzslglwo4)

[Table S4. Regression-estimated impact of seven interventions on the total number of one-time transfers to savings, average size of transfers to savings, and maximum transfer to savings. 21](#_kemicw9cskv)

[Table S5. Regression-estimated impact of seven interventions on whether a customer made any one-time transfers into a savings account from another account at the bank. 22](#_2g9kg7gv416t)

[Table S6. Regression-estimated impact of seven interventions on whether a customer made any recurring transfers into a savings account from another account at the bank. 24](#_yb9nm27sg2vd)

[Table S7. Regression-estimated impact of seven interventions on the change in a customer’s total savings balances during a given month of our two-month intervention or the three months post-intervention. 26](#_1vu4hmid90lh)

[Table S8. Means and standard deviations during a given month of our two-month intervention of customers’ (1) change in total monthly savings balances and (2) monthly transfers into savings among those in the business-as-usual control condition. 28](#_uuu32xiw1kdh)

[Table S9. Mean and standard deviation of the change in total savings balances during a given month of our two-month intervention for customers in the business-as-usual control condition. 29](#_7e8g47ykya1n)

[Table S10. Clicks on links in our emails by megastudy condition. 30](#_aetuxwjnd0jw)

[Table S11. Summary statistics for the overall sample and balance by condition in the total number of non-intervention emails sent to customers by our banking partner during our study. 31](#_wftjs9jxtr3w)

#

# 1. Definition of one-time and recurring transfers

One-time transfers into savings accounts tracked by our bank partner fall into two categories:

1. transfers from checking accounts and
2. transfers from other savings accounts.

Our one-time transfer outcome variable consists of only transfers of type (1) from checking accounts, which represent 98.33% of all one-time transfers into savings accounts per month because transferring money into a savings account from another savings account is not a way of increasing net savings.

Recurring transfers into savings accounts tracked by our bank partner fell into three categories:

1. transfers from checking accounts,
2. transfers from other savings accounts, and
3. transfers from an unknown internal source.

Our recurring transfers outcome variable again consists of only transfers of type (1) from checking accounts. Such transfers represent 99.33% of all recurring transfers into savings accounts per month. We focus on these transfers because, as noted above, (1) transferring money into a savings account from another savings account is not a way of increasing net savings and (2) transfers from unknown sources are rare (0.47% of cases per month), and we cannot verify that those sources are not another savings account.

#

# 2. Heterogeneity analyses

To explore possible heterogeneous treatment effects, we re-ran several of our main regression models (specifically, Table 4, Model 2; Table 5, Model 2; and Table 6, Models 2, 4, 6, and 8) with new interaction terms of interest entered into the model. The possible sources of heterogeneity we explored were as follows:

1. Balance in auto-loan
2. Indicator for having an auto-loan
3. Amount of money transacted at one of bank’s financial centers
4. Number of times money transacted at one of bank’s financial centers
5. Indicator for having money transacted at one of bank’s financial centers
6. Brokerage balance
7. Indicator for having a brokerage account with the bank
8. Balance of certificate of deposits or individual retirement accounts
9. Number of certificate of deposits or individual retirement accounts
10. Checking balance amount
11. Indicator for having a credit card account
12. Indicator for having an active credit card account
13. Balance of credit card accounts
14. Number of credit card accounts
15. Customer age
16. Number of months for having at least one account with the bank
17. Digital banking session count in the prior 90 days
18. Indicator for using a bank-sponsored digital goal setting tool
19. Home equity loan 3 month average balance amount
20. 3 month average revenue for home equity loan
21. Home equity loan balance amount
22. Indicator for having home equity loan
23. Number of home equity loan accounts
24. Customer income
25. Number of mobile banking session in the prior 90 days
26. 3 month average revenue for mortgage
27. Balance for mortgage accounts
28. Indicator for having a mortgage account
29. Number of mortgage accounts
30. Distance to the nearest banking center (miles)
31. Number of online banking session in the prior 90 days
32. Indicator for using a bank-sponsored round-up to the nearest dollar program
33. 3 month average savings account balance
34. Savings account balance
35. Number of savings accounts
36. Aggregate banking session count in the prior 90 days
37. Indicator for using the bank’s virtual financial assistant in the month prior to the start of the intervention period

To explore each of these 37 potential moderators effects, we added the following additional terms to our original regression models: (1) three-way interactions between the potential moderator, each of our seven indicators for assignment to our megastudy’s seven treatment conditions, and an indicator for whether the month in question occurred during our study’s two-month intervention period (March to April of 2022), and (2) an interaction between the potential moderator and an indicator for whether the month in question occurred during our study’s two-month intervention period (March to April of 2022). The key predictors of interest in our heterogeneity regressions were the aforementioned three-way interactions.

We first corrected for multiple comparisons using the Benjamini–Hochberg procedure, and then, to account for conducting 37 heterogeneity analyses, we also corrected for multiple comparisons using the Benjamini–Yekutieli procedure (Benjamini & Yekutieli, 2001). No results were significant after applying these corrections (all p’s > 0.718).

#

# 3. Post-intervention analyses

To explore possible durable treatment effects in the one, two and three-months post-intervention, we re-ran all of our main regression models (specifically, Table 4, Models 1 and 2; Table 5, Models 1 and 2; and Table 6, Models 1 through 8) with new terms of interest entered into the model. Specifically, we employed the same regression framework as in our aforementioned primary analyses but added (1) separate indicators to our models for observations from one month post-intervention, two months post-intervention and three months post-intervention and (2) interactions between each of these three new indicators and all treatment indicators (see Tables S5, S6, and S7).

# 4. Robustness check: Customers who did not have accounts in the pre-intervention period

In our megastudy, 10.68% of customers did not have a savings account with the bank at some point between April 2021 and November 2021. However, all customers had a savings account in the 90 days pre-intervention (from December 2021 to February 2022) because this was a prerequisite for study inclusion. In our manuscript, when analyzing customers’ change in savings balances (see Table 6), we simply do not include observations for customers during any pre-intervention month when they didn’t have a savings account with the bank. In analyses of one-time and recurring transfers, however, observations for customers without bank accounts are always included (but with zero transfers during months when they did not have a savings account). As a robustness check, we re-ran the analyses of one-time and recurring transfers in Tables 4 and 5 dropping observations in pre-intervention months when customers did not have a savings account with the bank. Our results are meaningfully unchanged when we make this adjustment.

# 5. Robustness check: Dropping customers with shared accounts

Prior to randomization, but after identifying a pool of approximately 2 million customers meeting our inclusion criteria, the bank de-duplicated the sample of customers who share accounts with other customers in the sample. This process involved assigning a randomly generated ID number to each customer and sorting them by address. If multiple customers shared the same address, the customer with the highest randomly generated ID was removed. However, when we received the data, we found that approximately 2.80% of customers in the sample shared at least one account with another customer in the sample from the start of the pre-intervention period through February 28, 2022. Among these 53,845 customers, 87.56% were assigned to different conditions.

Since our primary analysis is intent-to-treat, we did not exclude any customers from the sample. To evaluate whether including these customers affected our results, we re-ran the models from Tables 4, 5, and 6 using two modified versions of our sample: (1) excluding all customers sharing an account with another customer in the megastudy at any point in the pre-intervention or intervention period, and (2) excluding only those sharing an account with someone assigned to a different megastudy condition at any point in the pre-intervention or intervention period. Our key results are meaningfully unchanged when we make these adjustments.

# 6. Figures

## Figure S1. Illustration of the emails customers received if they were assigned to Intervention 1: Weekly Savings Reminders.

##
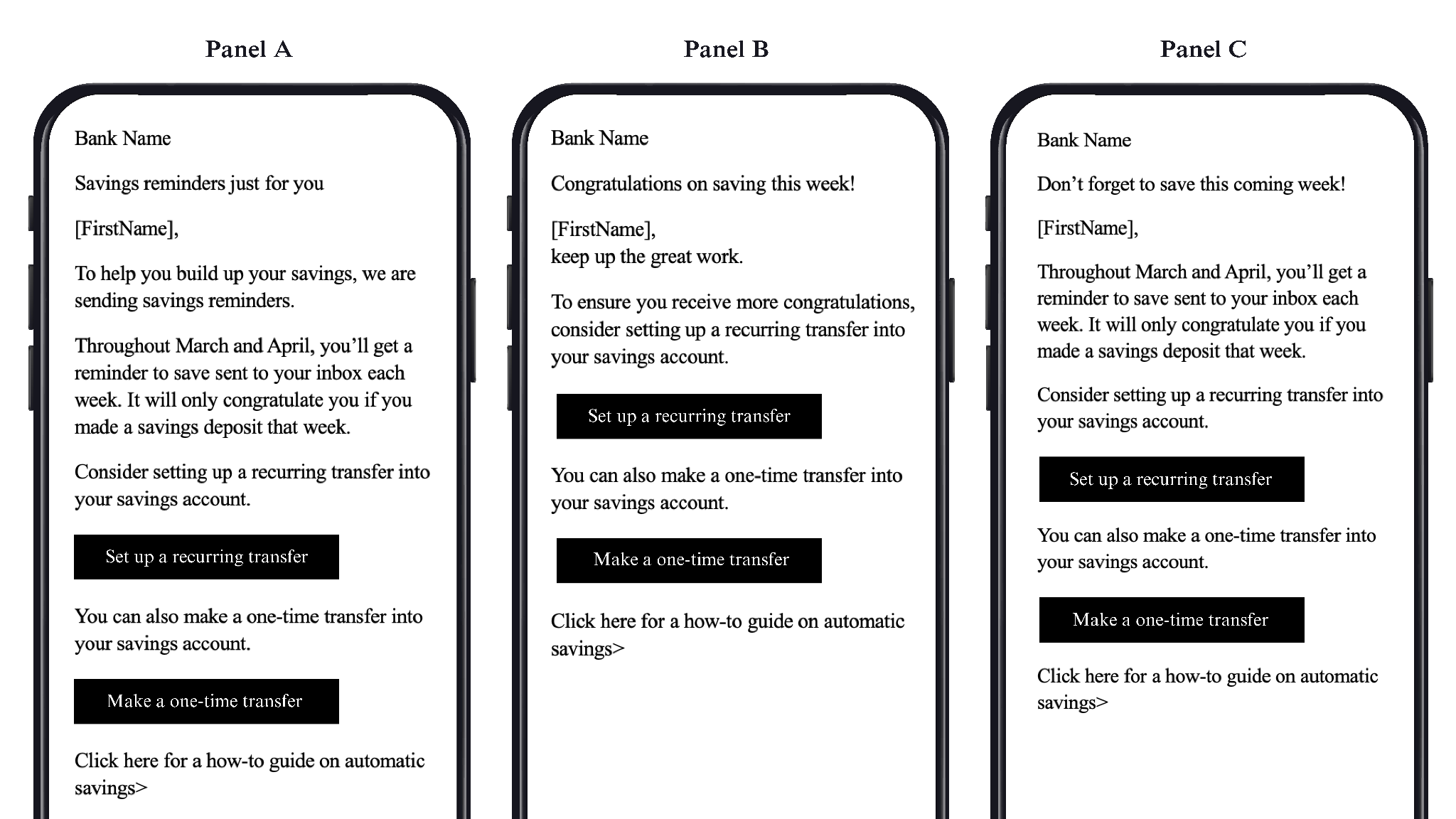


*Note:* Panel A shows a stripped down version of the initial email customers in this condition were sent on the 1^st^ of each intervention month introducing them to savings reminders. Panel B shows a stripped down version of the emails customers received on Friday of each week of the intervention period if they had made a savings deposit of any amount in the prior seven days. Panel C shows a stripped down version of the emails customers received on Friday of each week of the intervention period if they had not made a savings deposit in the prior seven days. All graphics and formatting except for buttons customers were encouraged to click have been removed at the request of our bank partner to preserve their confidentiality.

##

## Figure S2. Illustration of the emails customers received if they were assigned to Intervention 2: Weekly Savings Reminders with Wisdom Boxes.


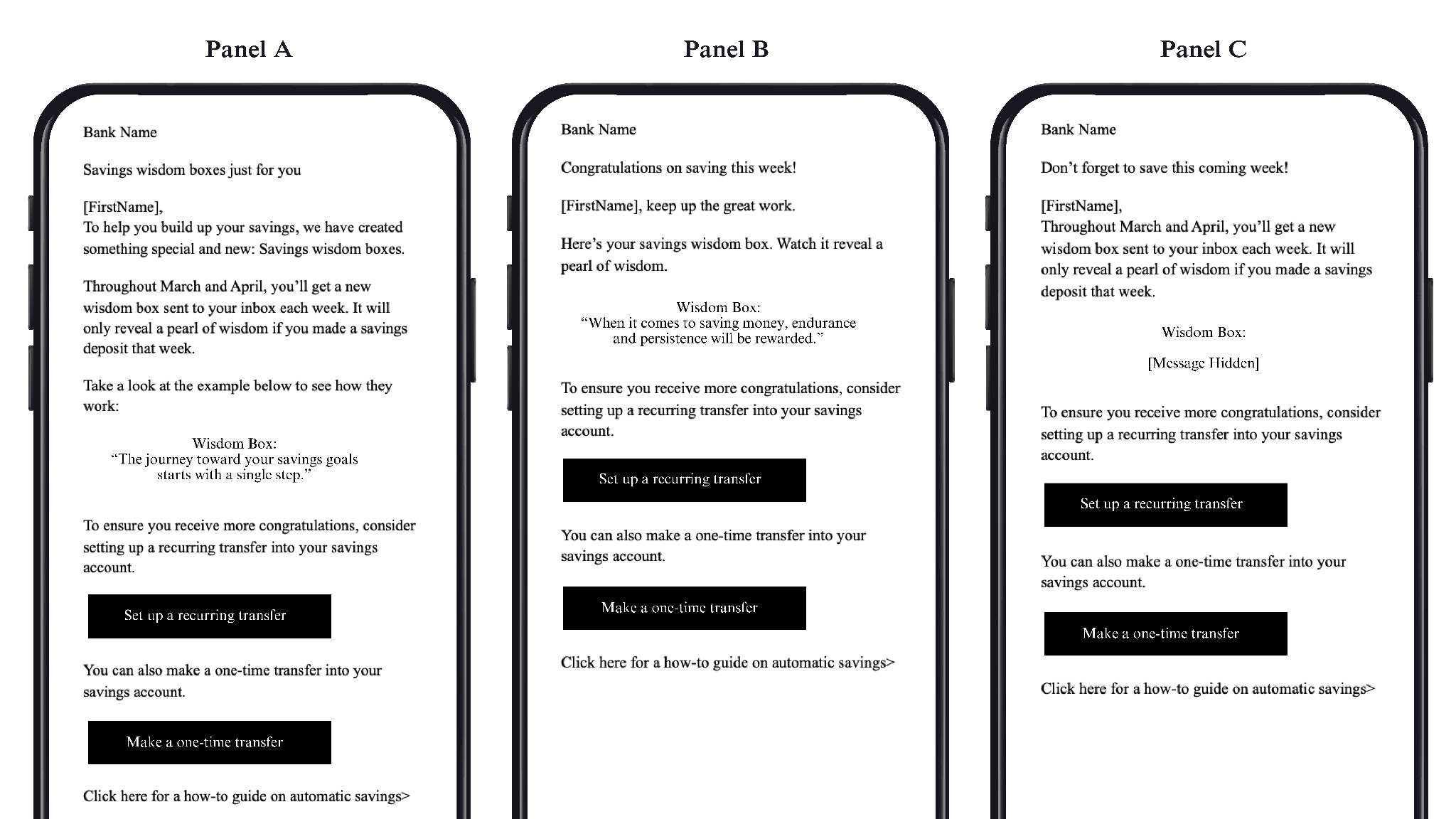


*Note:* Panel A shows a stripped down version of the initial email customers in this condition were sent on the 1^st^ of each intervention month introducing them to savings wisdom boxes. Panel B shows a stripped down version of the emails customers received on Friday of each week of the intervention period if they had made a savings deposit of any amount in the prior seven days. Panel C shows a stripped down version of the emails customers received on Friday of each week of the intervention period if they had not made a savings deposit in the prior seven days. All graphics and formatting except for buttons customers were encouraged to click have been removed at the request of our bank partner to preserve their confidentiality.

## Figure S3. Illustration of the emails customers received if they were assigned to Intervention 3: Monthly Random Savings Reminders.


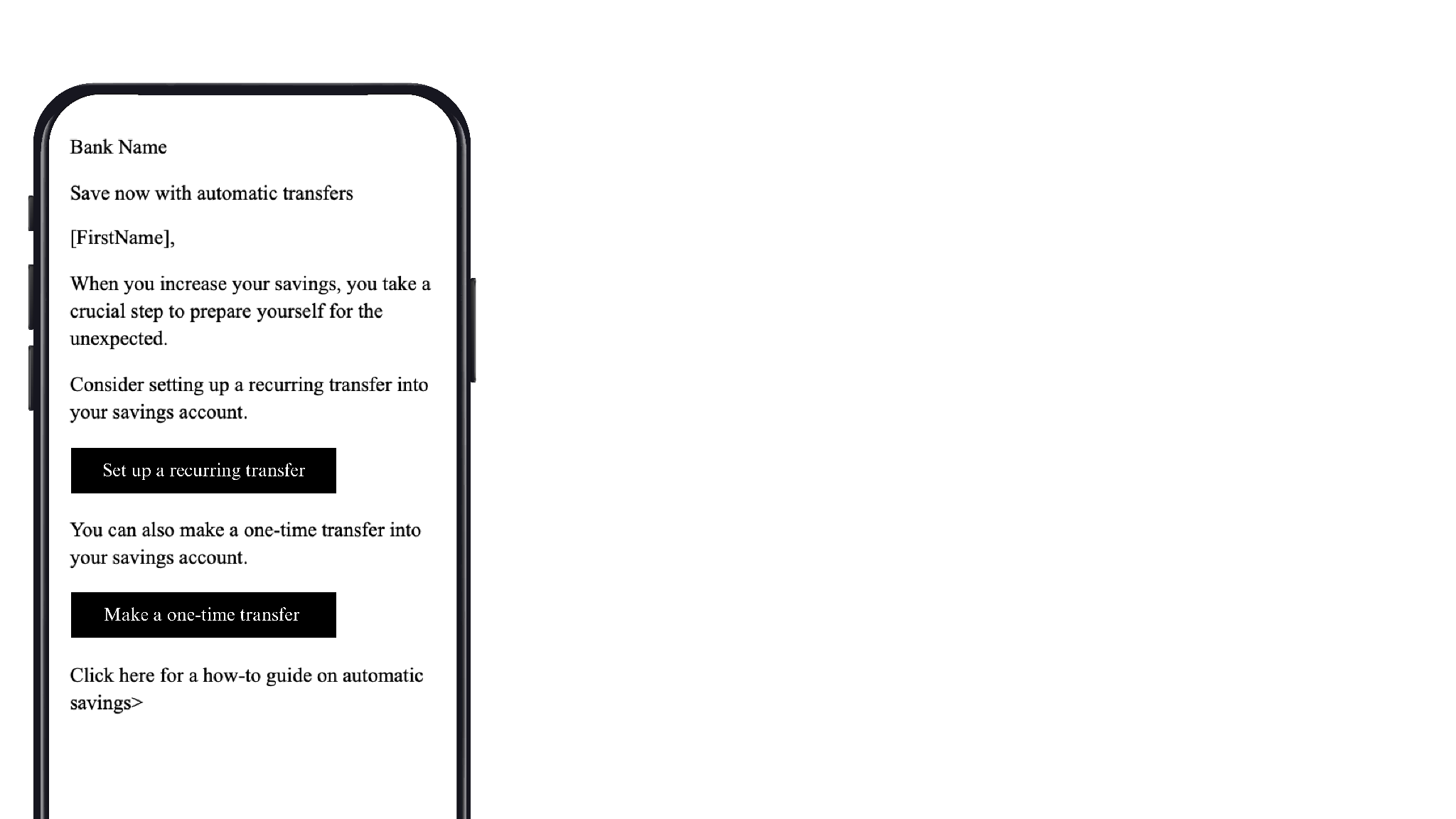


*Note:* All graphics and formatting except for buttons customers were encouraged to click have been removed at the request of our bank partner to preserve their confidentiality.

##

## Figure S4. Illustration of the emails customers received if they were assigned to Intervention 4: Monthly Reminders to Save Last.

**
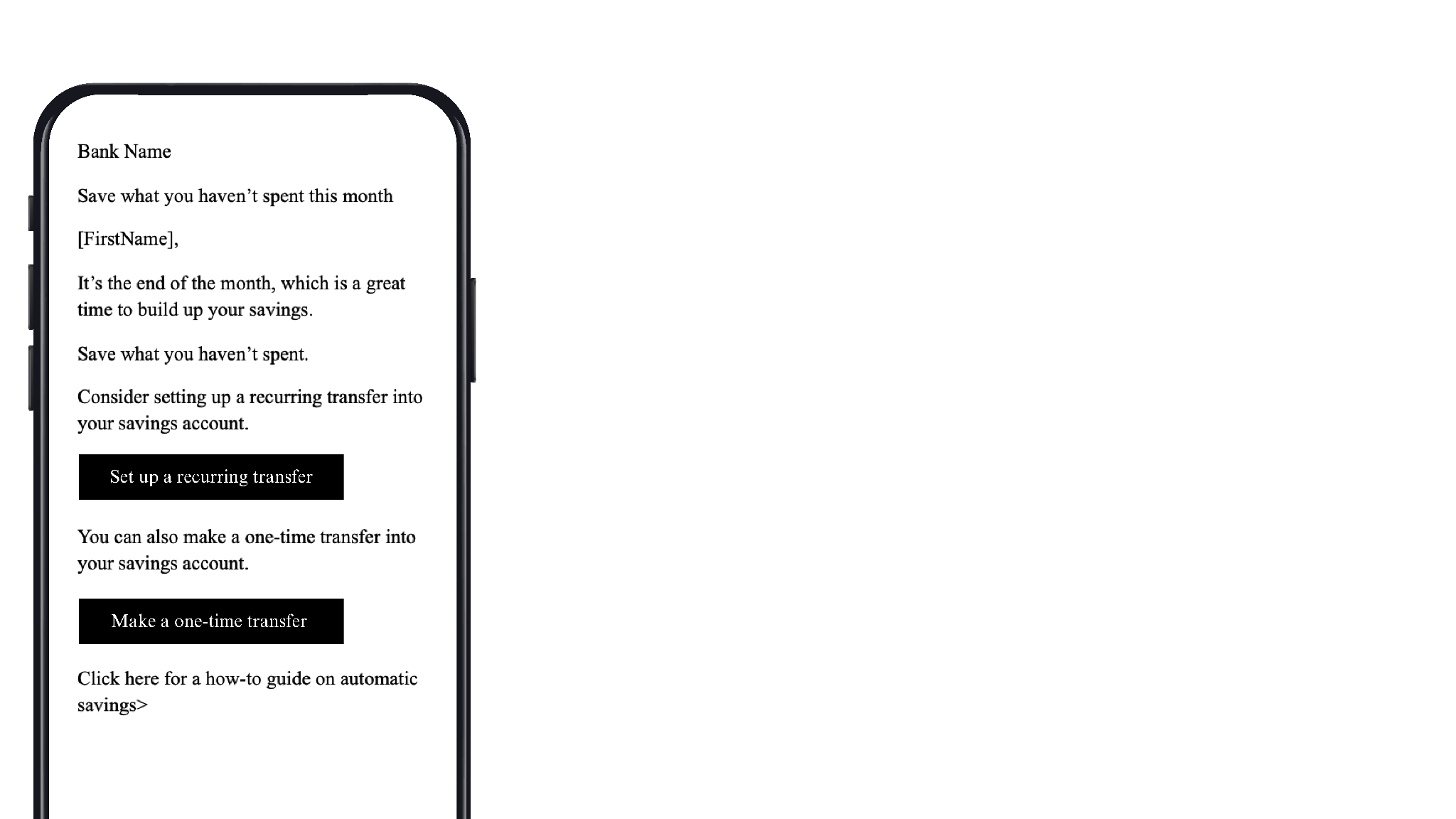
**

*Note:* All graphics and formatting except for buttons customers were encouraged to click have been removed at the request of our bank partner to preserve their confidentiality.

##

## Figure S5. Illustration of the emails customers received if they were assigned to Intervention 5: Monthly Reminders to Save First.


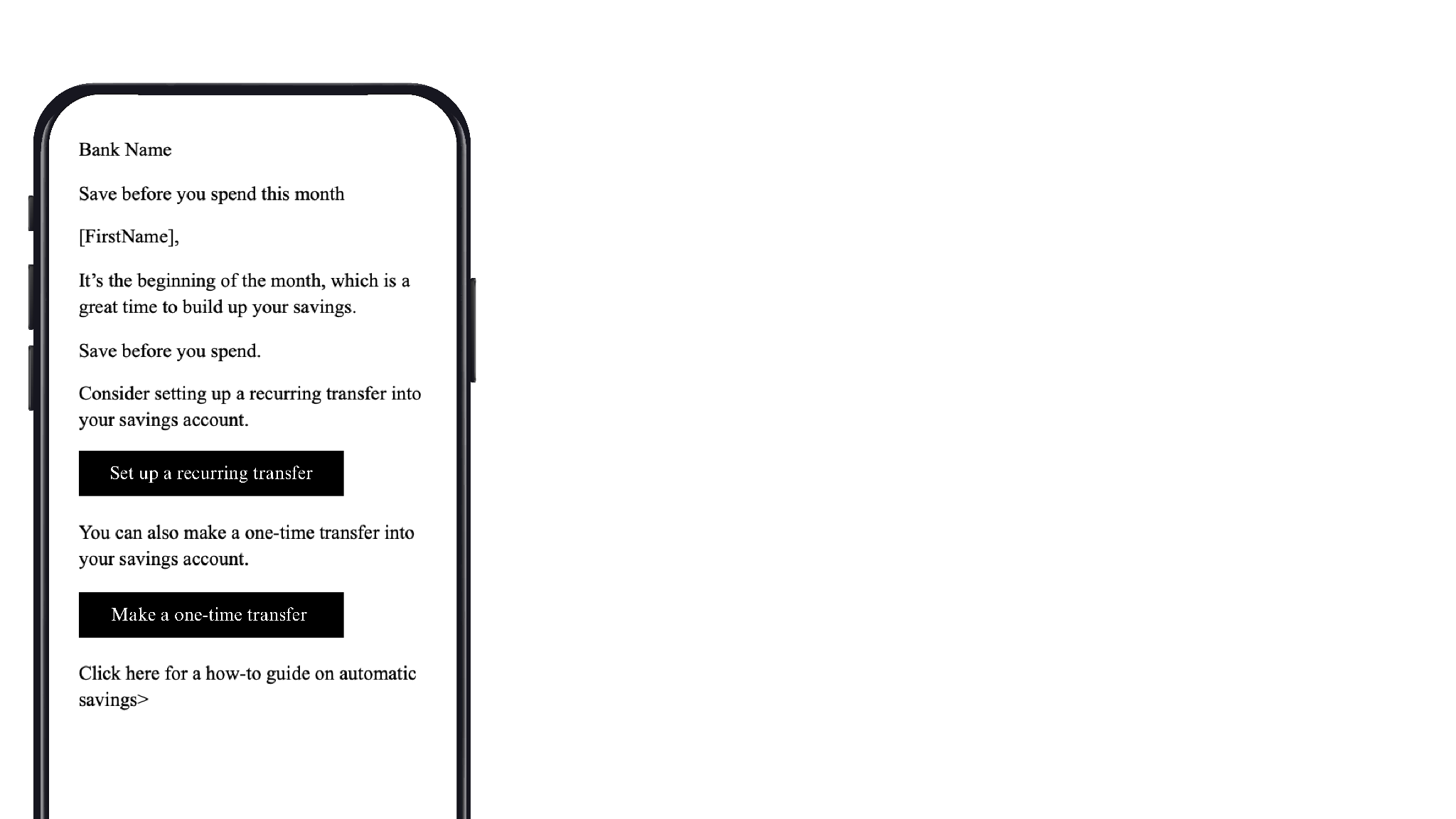


*Note:* All graphics and formatting except for buttons customers were encouraged to click have been removed at the request of our bank partner to preserve their confidentiality.

##

## Figure S6. Illustration of the emails customers received if they were assigned to Intervention 6: Monthly Implicit Deposit-Triggered Savings Reminders.


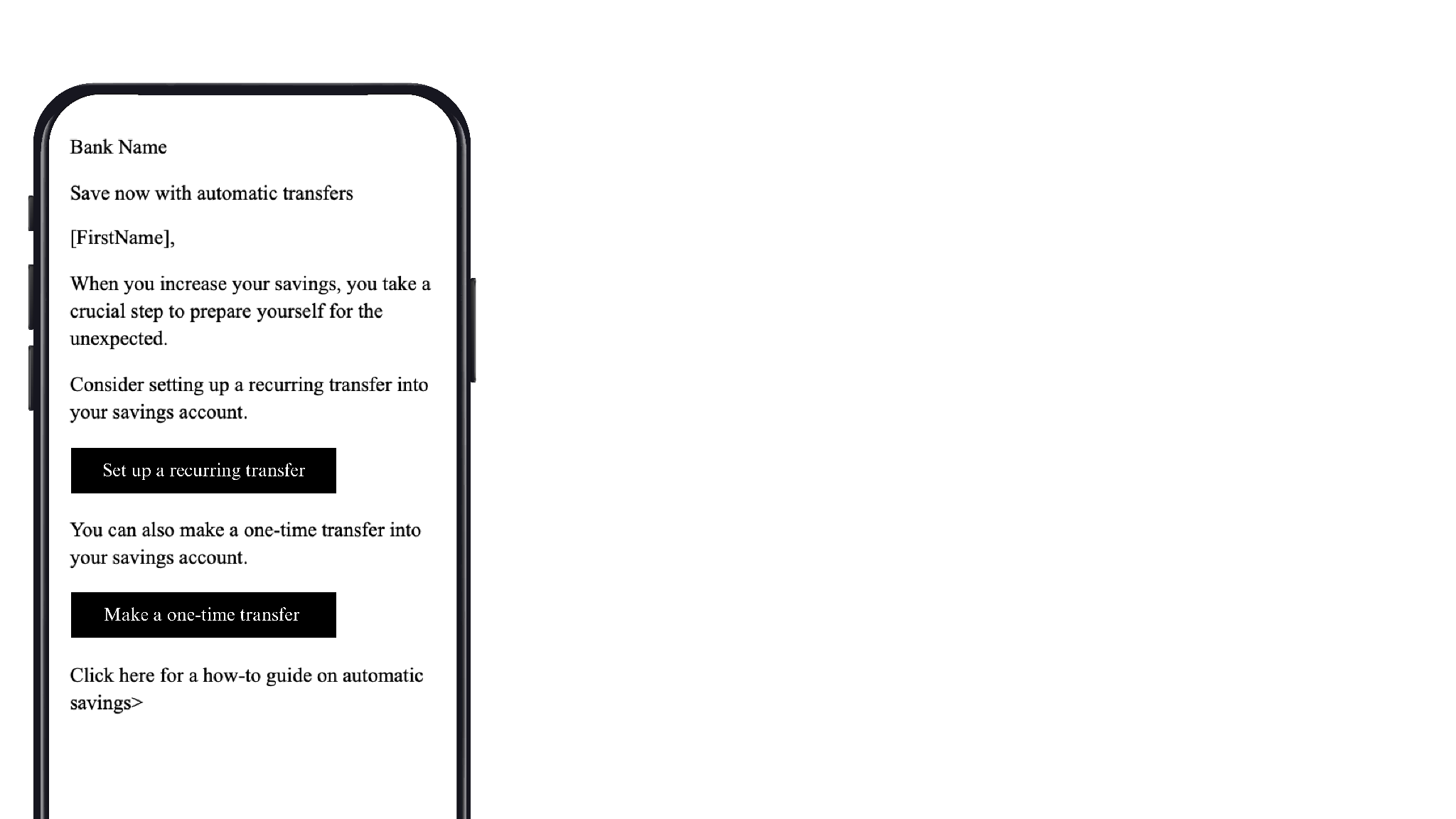


*Note:* All graphics and formatting except for buttons customers were encouraged to click have been removed at the request of our bank partner to preserve their confidentiality.

##

## Figure S7. Illustration of the emails customers received if they were assigned to Intervention 7: Monthly Explicit Deposit-Triggered Savings Reminders.


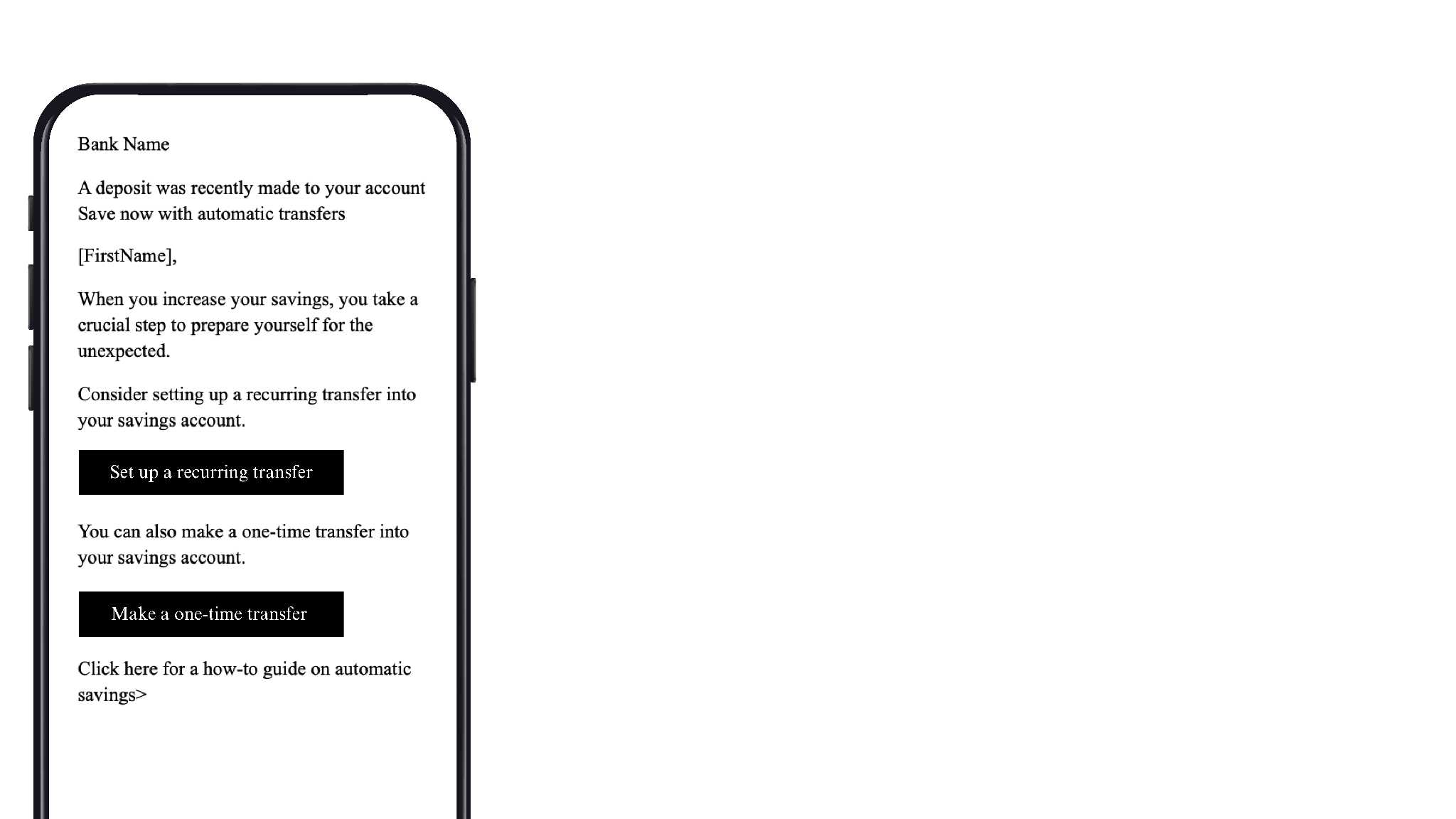


*Note:* All graphics and formatting except for buttons customers were encouraged to click have been removed at the request of our bank partner to preserve their confidentiality.

## Figure S8. Histograms depicting the distribution of changes in customers’ total savings balances per month during our two month intervention period and the eleven months prior: without trimming outliers showing bins of size $10,000 (Panel A), winsorizing data at the 1st and 99th percentiles showing bins of size $100 (Panel B), winsorizing data at the 5th and 95th percentiles showing bins of size $100 (Panel C), winsorizing data at the 10th and 90th percentiles showing bins of size $100 (Panel D), and using an inverse hyperbolic sine transformation of data showing bins of size 1 unit (Panel E).


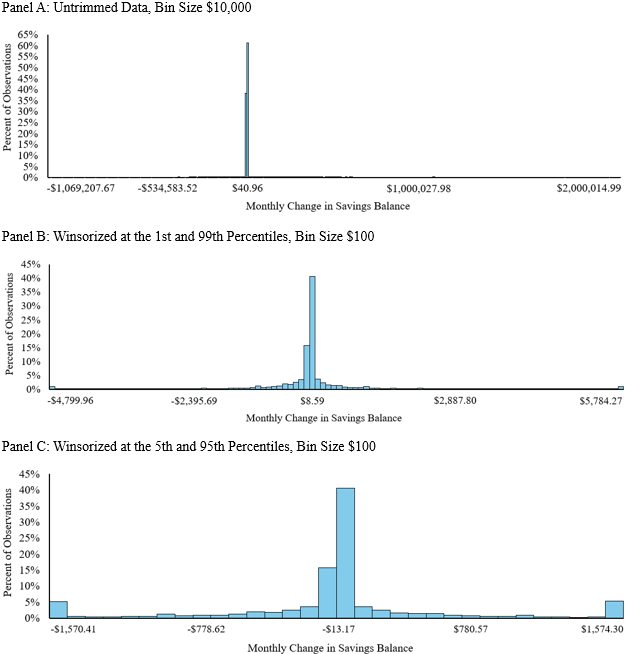


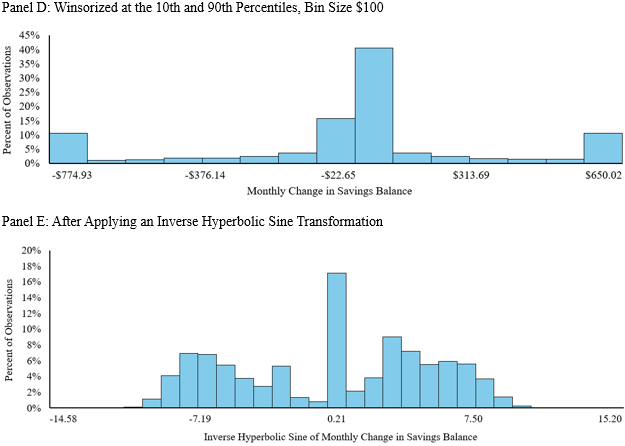


*Note:* Each histogram displays the minimum, mean, and maximum values of the distribution on the x-axis.

# 7. Tables

## Table S1. Test of ranks of interventions.

|  | DV: Made Any One-time Transfers to Savings | |  | DV: Δ in Monthly Savings  Winsorized at the 1st and 99th Pctls | |  | DV: Δ in Monthly Savings  Winsorized at the 5th and 95th Pctls | |  | DV: Δ in Monthly Savings  Winsorized at the 10th and 90th Pctls | |  | DV: IHS(Δ in Monthly Savings) | |
| --- | --- | --- | --- | --- | --- | --- | --- | --- | --- | --- | --- | --- | --- | --- |
|  | Model 1 | |  | Model 2 | |  | Model 3 | |  | Model 4 | |  | Model 5 | |
|  | *β* | Lower  Bound |  | *β* | Lower  Bound |  | *β* | Lower  Bound |  | *β* | Lower  Bound |  | *β* | Lower  Bound |
| Intervention 1: Savings Reminders | 0.125 | 1 |  | 3.852 | 1 |  | 3.596 | 1 |  | 2.326 | 1 |  | 0.034 | 1 |
|  | (0.026) |  |  | (2.772) |  |  | (1.412) |  |  | (0.803) |  |  | (0.011) |  |
| Intervention 2: Savings Wisdom Boxes | 0.086 | 1 |  | 1.664 | 1 |  | 1.535 | 1 |  | 1.048 | 1 |  | 0.014 | 1 |
|  | (0.026) |  |  | (2.758) |  |  | (1.408) |  |  | (0.802) |  |  | (0.011) |  |
| Intervention 3: Random Reminders | 0.050 | 2 |  | 3.603 | 1 |  | 2.803 | 1 |  | 1.558 | 1 |  | 0.017 | 1 |
|  | (0.026) |  |  | (2.761) |  |  | (1.408) |  |  | (0.802) |  |  | (0.011) |  |
| Intervention 4: Save Last | 0.040 | 3 |  | -0.283 | 1 |  | 0.401 | 1 |  | 0.432 | 2 |  | 4.924E-04 | 2 |
|  | (0.026) |  |  | (2.764) |  |  | (1.409) |  |  | (0.802) |  |  | (0.011) |  |
| Intervention 5: Save First | 0.036 | 3 |  | 0.203 | 1 |  | 2.127 | 1 |  | 1.684 | 1 |  | 0.016 | 1 |
|  | (0.026) |  |  | (2.762) |  |  | (1.407) |  |  | (0.801) |  |  | (0.011) |  |
| Intervention 6: Deposit-Triggered Implicit | 0.024 | 3 |  | 1.396 | 1 |  | 1.336 | 1 |  | 0.705 | 1 |  | 0.008 | 1 |
|  | (0.026) |  |  | (2.766) |  |  | (1.406) |  |  | (0.801) |  |  | (0.011) |  |
| Intervention 7: Deposit-Triggered Explicit | 0.016 | 3 |  | 1.398 | 1 |  | 1.486 | 1 |  | 0.993 | 1 |  | 0.009 | 1 |
|  | (0.026) |  |  | (2.767) |  |  | (1.410) |  |  | (0.803) |  |  | (0.011) |  |
| Observations | 26,960,990 | |  | 24,124,139 | |  | 24,124,139 | |  | 24,124,139 | |  | 24,124,139 | |

*Note*: This table presents the estimated betas and standard errors from five variants of our main regression models (specifically, Table 4, Model 2; Table 6, Model 2; Table 6, Model 4; Table 6, Model 6; and Table 6, Model 8). The number in the column “Lower Bound” is the lowest value in each condition’s one-sided 95% confidence set of its true rank, based on the set of seven regression-estimated intervention indicators. Each model uses a slightly different outcome variable, as labeled above. Each model includes 1,925,785 customers. Model 1 includes 14 months of observations per customer, whereas Models 2-5 include 13 months of observations per customer. The results of Model 1 indicate that intervention 1 (Savings Reminders) and intervention 2 (Savings Wisdom Boxes) are the only interventions for which we cannot reject the null hypothesis (at 95% confidence) that they are the true best-performing interventions when the outcome of interest is whether customers made any one-time transfers into their savings account from another account at the bank during a given month. The results of Model 2 indicate that for all the interventions we cannot reject the null hypothesis (at 95% confidence) that they are the true best-performing intervention when the monthly change in savings balance data is winsorized at the 1st and 99th percentiles. The results for Model 3 indicate that for all the interventions we cannot reject the null hypothesis (at 95% confidence) that they are the true best-performing intervention when the monthly change in savings balance data is winsorized at the 5th and 95th percentiles. The results for Model 4 indicate that interventions 1, 2, 3, 5, 6, and 7 are the only interventions for which we cannot reject the null hypothesis (at 95% confidence) that they are the true best-performing intervention when the monthly change in savings balance data is winsorized at the 10th and 90th percentiles. The results for Model 5 show that interventions 1, 2, 3, 5, and 7 are the only interventions for which we cannot reject the null hypothesis (at 95% confidence) that they are the true best-performing intervention when the monthly change in savings balance data is transformed using the inverse hyperbolic sine.

## Table S2. Wald tests comparing (1) the regression-estimated impacts of each of our megastudy’s intervention conditions on customers’ monthly likelihood of making a one-time transfer into savings from another bank account with (2) the regression-estimated impact of our megastudy’s intervention with the largest regression-estimated impact (Intervention 1. Savings Reminders) on customers’ monthly likelihood of making a one-time transfer into savings from another bank account.

|  | F | *P-value* | *BH-Adjusted P-value* |
| --- | --- | --- | --- |
| H0: (Intervention 1. Savings Reminders) = (Intervention 2. Savings Wisdom Boxes) | 2.139 | 0.144 | 0.144 |
| H0: (Intervention 1. Savings Reminders) = (Intervention 3. Random Reminders) | 7.858 | 0.005 | 0.006 |
| H0: (Intervention 1. Savings Reminders) = (Intervention 4. Save Last) | 10.231 | 0.001 | 0.002 |
| H0: (Intervention 1. Savings Reminders) = (Intervention 5. Save First) | 11.063 | 0.001 | 0.002 |
| H0: (Intervention 1. Savings Reminders) = (Intervention 6. Deposit-Triggered Implicit) | 14.251 | <0.001 | <0.001 |
| H0: (Intervention 1. Savings Reminders) = (Intervention 7. Deposit-Triggered Explicit) | 16.607 | <0.001 | <0.001 |

*Note:* The Wald tests presented in this table were performed following the estimation of the regression model in Table 4, Model 2. P-values are reported both unadjusted and adjusted for multiple comparisons using the Benjamini-Hochberg (BH) procedure.

## Table S3. Wald tests comparing (1) the regression-estimated impacts of each of our megastudy’s intervention conditions on customers’ monthly change in savings balances with (2) the regression-estimated impact of our megastudy’s intervention with the largest regression-estimated impact (Intervention 1. Savings Reminders) on customers’ monthly change in savings balances.

| Table 6, Model 4 | F | *P* | *BH-Adjusted P* |
| --- | --- | --- | --- |
| H0: (Intervention 1. Savings Reminders) = (Intervention 2. Savings Wisdom Boxes) | 2.131 | 0.144 | 0.217 |
| H0: (Intervention 1. Savings Reminders) = (Intervention 3. Random Reminders) | 0.315 | 0.574 | 0.574 |
| H0: (Intervention 1. Savings Reminders) = (Intervention 4. Save Last) | 5.118 | 0.024 | 0.142 |
| H0: (Intervention 1. Savings Reminders) = (Intervention 5. Save First) | 1.085 | 0.298 | 0.357 |
| H0: (Intervention 1. Savings Reminders) = (Intervention 6. Deposit-Triggered Implicit) | 2.569 | 0.109 | 0.217 |
| H0: (Intervention 1. Savings Reminders) = (Intervention 7. Deposit-Triggered Explicit) | 2.229 | 0.135 | 0.217 |
|  |  |  |  |
| Table 6, Model 6 | F | *P* | *BH-Adjusted P* |
| H0: (Intervention 1. Savings Reminders) = (Intervention 2. Savings Wisdom Boxes) | 2.534 | 0.111 | 0.167 |
| H0: (Intervention 1. Savings Reminders) = (Intervention 3. Random Reminders) | 0.915 | 0.339 | 0.407 |
| H0: (Intervention 1. Savings Reminders) = (Intervention 4. Save Last) | 5.558 | 0.018 | 0.110 |
| H0: (Intervention 1. Savings Reminders) = (Intervention 5. Save First) | 0.640 | 0.424 | 0.424 |
| H0: (Intervention 1. Savings Reminders) = (Intervention 6. Deposit-Triggered Implicit) | 4.086 | 0.043 | 0.130 |
| H0: (Intervention 1. Savings Reminders) = (Intervention 7. Deposit-Triggered Explicit) | 2.752 | 0.097 | 0.167 |
|  |  |  |  |
| Table 6, Model 8 | F | *P* | *BH-Adjusted P* |
| H0: (Intervention 1. Savings Reminders) = (Intervention 2. Savings Wisdom Boxes) | 3.110 | 0.078 | 0.117 |
| H0: (Intervention 1. Savings Reminders) = (Intervention 3. Random Reminders) | 2.188 | 0.139 | 0.139 |
| H0: (Intervention 1. Savings Reminders) = (Intervention 4. Save Last) | 8.675 | 0.003 | 0.019 |
| H0: (Intervention 1. Savings Reminders) = (Intervention 5. Save First) | 2.648 | 0.104 | 0.124 |
| H0: (Intervention 1. Savings Reminders) = (Intervention 6. Deposit-Triggered Implicit) | 5.439 | 0.020 | 0.054 |
| H0: (Intervention 1. Savings Reminders) = (Intervention 7. Deposit-Triggered Explicit) | 4.886 | 0.027 | 0.054 |

*Note:* The Wald tests presented in this table were performed following the estimation of our main regression models presented in Table 6, Models 4, 6, and 8. P-values are reported both unadjusted and adjusted for multiple comparisons using the Benjamini-Hochberg (BH) procedure.

##

## Table S4. Regression-estimated impact of our megastudy’s seven intervention conditions on the total number of one-time transfers to savings (Models 1-2), average size of transfers to savings (Models 3-4), and maximum transfer to savings during a given month of our two-month intervention (Models 5-6); either pooling all intervention conditions (Models 1, 3, and 5), or by intervention condition (Models 2, 4, and 6).

|  | DV: Total Number of One-time Transfers to Savings | | | | |  | DV: Average Size of Transfer to Savings | | | | |  | DV: Maximum Transfer to Savings | | | | |
| --- | --- | --- | --- | --- | --- | --- | --- | --- | --- | --- | --- | --- | --- | --- | --- | --- | --- |
|  | Model 1 | |  | Model 2 | |  | Model 3 | |  | Model 4 | |  | Model 5 | |  | Model 6 | |
|  | *β* | *P-value* |  | *β* | *BH-Adjusted P-value* |  | *β* | *P-value* |  | *β* | *BH-Adjusted P-value* |  | *β* | *P-value* |  | *β* | *BH-Adjusted P-value* |
| (Assigned Any Intervention) x (Intervention Period) | -1.578E-04 | 0.921 |  |  |  |  | 0.278 | 0.516 |  |  |  |  | 0.383 | 0.402 |  |  |  |
|  | (0.002) |  |  |  |  |  | (0.428) |  |  |  |  |  | (0.457) |  |  |  |  |
| (Intervention 1. Savings Reminders) x (Intervention Period) |  |  |  | 0.003*** | < 0.001 |  |  |  |  | 0.159 | 0.932 |  |  |  |  | 0.206 | 0.877 |
|  |  |  |  | (0.001) |  |  |  |  |  | (0.573) |  |  |  |  |  | (0.604) |  |
| (Intervention 2. Savings Wisdom Boxes) x (Intervention Period) |  |  |  | 0.002* | 0.040 |  |  |  |  | -0.158 | 0.932 |  |  |  |  | 0.090 | 0.877 |
|  |  |  |  | (0.001) |  |  |  |  |  | (0.527) |  |  |  |  |  | (0.583) |  |
| (Intervention 3. Random Reminders) x (Intervention Period) |  |  |  | 0.001 | 0.410 |  |  |  |  | -0.004 | 0.994 |  |  |  |  | -0.096 | 0.877 |
|  |  |  |  | (0.001) |  |  |  |  |  | (0.575) |  |  |  |  |  | (0.595) |  |
| (Intervention 4. Save Last) x (Intervention Period) |  |  |  | 0.001 | 0.129 |  |  |  |  | 0.288 | 0.932 |  |  |  |  | 0.388 | 0.877 |
|  |  |  |  | (0.001) |  |  |  |  |  | (0.678) |  |  |  |  |  | (0.704) |  |
| (Intervention 5. Save First) x (Intervention Period) |  |  |  | 0.001 | 0.410 |  |  |  |  | 0.134 | 0.932 |  |  |  |  | 0.277 | 0.877 |
|  |  |  |  | (0.001) |  |  |  |  |  | (0.525) |  |  |  |  |  | (0.555) |  |
| (Intervention 6. Deposit-Triggered Implicit) x (Intervention Period) |  |  |  | 0.001 | 0.424 |  |  |  |  | 0.960 | 0.932 |  |  |  |  | 1.321 | 0.877 |
|  |  |  |  | (0.001) |  |  |  |  |  | (0.818) |  |  |  |  |  | (1.021) |  |
| (Intervention 7. Deposit-Triggered Explicit) x (Intervention Period) |  |  |  | -0.010 | 0.410 |  |  |  |  | 0.569 | 0.932 |  |  |  |  | 0.498 | 0.877 |
|  |  |  |  | (0.010) |  |  |  |  |  | (0.706) |  |  |  |  |  | (0.724) |  |
| F-statistic for F-test of equality across 7 study conditions |  |  |  | 2.452 | 0.023 |  |  |  |  | 0.444 | 0.850 |  |  |  |  | 0.384 | 0.890 |
| Observations | 26,960,990 | |  | 26,960,990 | |  | 26,960,990 | |  | 26,960,990 | |  | 26,960,990 | |  | 26,960,990 | |
| Customer Fixed Effects | 1,925,785 | |  | 1,925,785 | |  | 1,925,785 | |  | 1,925,785 | |  | 1,925,785 | |  | 1,925,785 | |
| Month Fixed Effects | 14 | |  | 14 | |  | 14 | |  | 14 | |  | 14 | |  | 14 | |
| R-Squared | 0.515 | |  | 0.515 | |  | 0.146 | |  | 0.146 | |  | 0.143 | |  | 0.143 | |

*Note:* This table reports the results of six ordinary least squares (OLS) regressions predicting the total number of one-time transfers to savings, average size of transfers to savings, and maximum size of transfers to savings. All models include 14 months of observations for each customer (from March of 2021 - April of 2022), and all models include customer fixed effects as well as month fixed effects. In Models 1, 3, and 5, the primary predictor variable is an interaction between an indicator for whether the observation month fell during the study’s two-month intervention period and an indicator for whether a customer was assigned to receive any of our megastudy’s seven intervention conditions. In Models 2, 4, and 6, the primary predictor variables are interactions between an indicator for whether the observation month fell during the study’s two-month intervention period and separate indicators for whether a customer was assigned to each of our megastudy’s seven intervention conditions. Standard errors reported in parentheses are clustered at the customer level, and p-values in Models 2, 4, and 6 are adjusted for multiple comparisons using the Benjamini-Hochberg procedure. * p < 0.05 ** p < 0.01 *** p < 0.001.

## Table S5. Regression-estimated impact of our megastudy’s seven intervention conditions on whether a customer made any one-time transfers into a savings account from another account at the bank during a given month of our two-month intervention period or the three months following, either pooling all intervention conditions (Model 1), or by intervention condition (Model 2).

|  |  | DV: Made Any One-time Transfers to Savings | | | | |
| --- | --- | --- | --- | --- | --- | --- |
|  |  | Model 1 | |  | Model 2 | |
| Time Period | Predictor Variable | *β* | *P-value* |  | *β* | *BH-Adjusted P-value* |
| During Intervention | (Assigned Any Intervention) x (Intervention-Period) | 0.054** | 0.006 |  |  |  |
|  |  | (0.019) |  |  |  |  |
|  | (Intervention 1. Savings Reminders) x (Intervention-Period) |  |  |  | 0.125*** | <0.001 |
|  |  |  |  |  | (0.026) |  |
|  | (Intervention 2. Savings Wisdom Boxes) x (Intervention-Period) |  |  |  | 0.086** | 0.003 |
|  |  |  |  |  | (0.026) |  |
|  | (Intervention 3. Random Reminders) x (Intervention-Period) |  |  |  | 0.050 | 0.129 |
|  |  |  |  |  | (0.026) |  |
|  | (Intervention 4. Save Last) x (Intervention-Period) |  |  |  | 0.040 | 0.211 |
|  |  |  |  |  | (0.026) |  |
|  | (Intervention 5. Save First) x (Intervention-Period) |  |  |  | 0.036 | 0.233 |
|  |  |  |  |  | (0.026) |  |
|  | (Intervention 6. Deposit-Triggered Implicit) x (Intervention-Period) |  |  |  | 0.024 | 0.418 |
|  |  |  |  |  | (0.026) |  |
|  | (Intervention 7. Deposit-Triggered Explicit) x (Intervention-Period) |  |  |  | 0.016 | 0.533 |
|  |  |  |  |  | (0.026) |  |
| 1 Month Post-Intervention | (Assigned Any Intervention) x (Post-Intervention Month 1) | 0.079** | 0.004 |  |  |  |
|  |  | (0.027) |  |  |  |  |
|  | (Intervention 1. Savings Reminders) x (Post-Intervention Month 1) |  |  |  | 0.138** | 0.001 |
|  |  |  |  |  | (0.037) |  |
|  | (Intervention 2. Savings Wisdom Boxes) x (Post-Intervention Month 1) |  |  |  | 0.105** | 0.009 |
|  |  |  |  |  | (0.036) |  |
|  | (Intervention 3. Random Reminders) x (Post-Intervention Month 1) |  |  |  | 0.044 | 0.258 |
|  |  |  |  |  | (0.036) |  |
|  | (Intervention 4. Save Last) x (Post-Intervention Month 1) |  |  |  | 0.104** | 0.009 |
|  |  |  |  |  | (0.036) |  |
|  | (Intervention 5. Save First) x (Post-Intervention Month 1) |  |  |  | 0.082* | 0.040 |
|  |  |  |  |  | (0.036) |  |
|  | (Intervention 6. Deposit-Triggered Implicit) x (Post-Intervention Month 1) |  |  |  | 0.007 | 0.842 |
|  |  |  |  |  | (0.036) |  |
|  | (Intervention 7. Deposit-Triggered Explicit) x (Post-Intervention Month 1) |  |  |  | 0.070 | 0.074 |
|  |  |  |  |  | (0.036) |  |
| 2 Months Post-Intervention | (Assigned Any Intervention) x (Post-Intervention Month 2) | 0.039 | 0.113 |  |  |  |
|  |  | (0.025) |  |  |  |  |
|  | (Intervention 1. Savings Reminders) x (Post-Intervention Month 2) |  |  |  | 0.076 | 0.091 |
|  |  |  |  |  | (0.033) |  |
|  | (Intervention 2. Savings Wisdom Boxes) x (Post-Intervention Month 2) |  |  |  | 0.073 | 0.091 |
|  |  |  |  |  | (0.033) |  |
|  | (Intervention 3. Random Reminders) x (Post-Intervention Month 2) |  |  |  | 0.059 | 0.129 |
|  |  |  |  |  | (0.033) |  |
|  | (Intervention 4. Save Last) x (Post-Intervention Month 2) |  |  |  | 0.065 | 0.109 |
|  |  |  |  |  | (0.033) |  |
|  | (Intervention 5. Save First) x (Post-Intervention Month 2) |  |  |  | 0.021 | 0.612 |
|  |  |  |  |  | (0.033) |  |
|  | (Intervention 6. Deposit-Triggered Implicit) x (Post-Intervention Month 2) |  |  |  | - 0.024 | 0.612 |
|  |  |  |  |  | (0.033) |  |
|  | (Intervention 7. Deposit-Triggered Explicit) x (Post-Intervention Month 2) |  |  |  | 0.004 | 0.913 |
|  |  |  |  |  | (0.033) |  |
| 3 Months Post-Intervention | (Assigned Any Intervention) x (Post-Intervention Month 3) | 0.038 | 0.187 |  |  |  |
|  |  | (0.029) |  |  |  |  |
|  | (Intervention 1. Savings Reminders) x (Post-Intervention Month 3) |  |  |  | 0.087 | 0.088 |
|  |  |  |  |  | (0.039) |  |
|  | (Intervention 2. Savings Wisdom Boxes) x (Post-Intervention Month 3) |  |  |  | 0.099 | 0.081 |
|  |  |  |  |  | (0.039) |  |
|  | (Intervention 3. Random Reminders) x (Post-Intervention Month 3) |  |  |  | 0.022 | 0.987 |
|  |  |  |  |  | (0.039) |  |
|  | (Intervention 4. Save Last) x (Post-Intervention Month 3) |  |  |  | 0.013 | 0.987 |
|  |  |  |  |  | (0.038) |  |
|  | (Intervention 5. Save First) x (Post-Intervention Month 3) |  |  |  | 0.006 | 0.987 |
|  |  |  |  |  | (0.039) |  |
|  | (Intervention 6. Deposit-Triggered Implicit) x (Post-Intervention Month 3) |  |  |  | - 0.001 | 0.987 |
|  |  |  |  |  | (0.039) |  |
|  | (Intervention 7. Deposit-Triggered Explicit) x (Post-Intervention Month 3) |  |  |  | 0.043 | 0.632 |
|  |  |  |  |  | (0.039) |  |
|  | Observations | 32,738,345 | |  | 32,738,345 | |
|  | Customer Fixed Effects | 1,925,785 | |  | 1,925,785 | |
|  | Month Fixed Effects | 17 | |  | 17 | |
|  | R-Squared | 0.899 | |  | 0.899 | |

*Note:* This table reports the results of two ordinary least squares (OLS) regressions predicting whether a given customer made any one-time transfers into their savings account at the bank in a given month. Both models include 17 months of observations for each customer (from March of 2021 - July of 2022), and both models include customer fixed effects as well as month fixed effects. In Model 1, the primary predictor variables are four interactions between an indicator for whether a customer was assigned to receive any of our megastudy’s seven intervention conditions and four indicators for the study’s two month intervention period and each of the three post-intervention months. In Model 2, the primary predictor variables are twenty-eight interactions between separate indicators for whether a customer was assigned to each of our megastudy’s seven intervention conditions and four indicators for the study’s two month intervention period and each of the three post-intervention months. Regression coefficients and standard errors have been multiplied by 100 to improve interpretability. Standard errors reported in parentheses are clustered at the customer level, and p-values in Model 2 are adjusted for multiple comparisons using the Benjamini-Hochberg procedure. * p < 0.05 ** p < 0.01 *** p < 0.001.

## Table S6. Regression-estimated impact of our megastudy’s seven intervention conditions on whether a customer made any recurring transfers into a savings account from another account at the bank during a given month of our two-month intervention period or the three months following, either pooling all intervention conditions (Model 1), or by intervention condition (Model 2).

|  |  | DV: Made Any Recurring Transfers to Savings | | | | |
| --- | --- | --- | --- | --- | --- | --- |
|  |  | Model 1 | |  | Model 2 | |
| Time Period |  | *β* | *P-value* |  | *β* | *BH-Adjusted P-value* |
| During Intervention | (Assigned Any Intervention) x (Intervention-Period) | -0.007 | 0.618 |  |  |  |
|  |  | (0.014) |  |  |  |  |
|  | (Intervention 1. Savings Reminders) x (Intervention-Period) |  |  |  | -0.015 | 0.751 |
|  |  |  |  |  | (0.019) |  |
|  | (Intervention 2. Savings Wisdom Boxes) x (Intervention-Period) |  |  |  | -0.017 | 0.751 |
|  |  |  |  |  | (0.019) |  |
|  | (Intervention 3. Random Reminders) x (Intervention-Period) |  |  |  | -0.004 | 0.969 |
|  |  |  |  |  | (0.019) |  |
|  | (Intervention 4. Save Last) x (Intervention-Period) |  |  |  | -0.028 | 0.751 |
|  |  |  |  |  | (0.019) |  |
|  | (Intervention 5. Save First) x (Intervention-Period) |  |  |  | 0.016 | 0.751 |
|  |  |  |  |  | (0.019) |  |
|  | (Intervention 6. Deposit-Triggered Implicit) x (Intervention-Period) |  |  |  | -0.002 | 0.969 |
|  |  |  |  |  | (0.019) |  |
|  | (Intervention 7. Deposit-Triggered Explicit) x (Intervention-Period) |  |  |  | 0.001 | 0.969 |
|  |  |  |  |  | (0.019) |  |
| 1 Month Post-Intervention | (Assigned Any Intervention) x (Post-Intervention Month 1) | -0.010 | 0.726 |  |  |  |
|  |  | (0.029) |  |  |  |  |
|  | (Intervention 1. Savings Reminders) x (Post-Intervention Month 1) |  |  |  | -0.010 | 0.795 |
|  |  |  |  |  | (0.038) |  |
|  | (Intervention 2. Savings Wisdom Boxes) x (Post-Intervention Month 1) |  |  |  | -0.019 | 0.795 |
|  |  |  |  |  | (0.038) |  |
|  | (Intervention 3. Random Reminders) x (Post-Intervention Month 1) |  |  |  | -0.013 | 0.795 |
|  |  |  |  |  | (0.038) |  |
|  | (Intervention 4. Save Last) x (Post-Intervention Month 1) |  |  |  | 0.026 | 0.795 |
|  |  |  |  |  | (0.038) |  |
|  | (Intervention 5. Save First) x (Post-Intervention Month 1) |  |  |  | -0.014 | 0.795 |
|  |  |  |  |  | (0.038) |  |
|  | (Intervention 6. Deposit-Triggered Implicit) x (Post-Intervention Month 1) |  |  |  | -0.028 | 0.795 |
|  |  |  |  |  | (0.038) |  |
|  | (Intervention 7. Deposit-Triggered Explicit) x (Post-Intervention Month 1) |  |  |  | -0.011 | 0.795 |
|  |  |  |  |  | (0.038) |  |
| 2 Months Post-Intervention | (Assigned Any Intervention) x (Post-Intervention Month 2) | -0.033 | 0.083 |  |  |  |
|  |  | (0.019) |  |  |  |  |
|  | (Intervention 1. Savings Reminders) x (Post-Intervention Month 2) |  |  |  | -0.057 | 0.062 |
|  |  |  |  |  | (0.026) |  |
|  | (Intervention 2. Savings Wisdom Boxes) x (Post-Intervention Month 2) |  |  |  | -0.057 | 0.062 |
|  |  |  |  |  | (0.026) |  |
|  | (Intervention 3. Random Reminders) x (Post-Intervention Month 2) |  |  |  | -0.040 | 0.201 |
|  |  |  |  |  | (0.026) |  |
|  | (Intervention 4. Save Last) x (Post-Intervention Month 2) |  |  |  | -0.057 | 0.062 |
|  |  |  |  |  | (0.026) |  |
|  | (Intervention 5. Save First) x (Post-Intervention Month 2) |  |  |  | -0.017 | 0.499 |
|  |  |  |  |  | (0.026) |  |
|  | (Intervention 6. Deposit-Triggered Implicit) x (Post-Intervention Month 2) |  |  |  | -0.026 | 0.438 |
|  |  |  |  |  | (0.026) |  |
|  | (Intervention 7. Deposit-Triggered Explicit) x (Post-Intervention Month 2) |  |  |  | 0.022 | 0.438 |
|  |  |  |  |  | (0.025) |  |
| 3 Months Post-Intervention | (Assigned Any Intervention) x (Post-Intervention Month 3) | -0.013 | 0.513 |  |  |  |
|  |  | (0.020) |  |  |  |  |
|  | (Intervention 1. Savings Reminders) x (Post-Intervention Month 3) |  |  |  | -0.032 | 0.420 |
|  |  |  |  |  | (0.027) |  |
|  | (Intervention 2. Savings Wisdom Boxes) x (Post-Intervention Month 3) |  |  |  | -0.039 | 0.373 |
|  |  |  |  |  | (0.027) |  |
|  | (Intervention 3. Random Reminders) x (Post-Intervention Month 3) |  |  |  | 0.001 | 0.979 |
|  |  |  |  |  | (0.027) |  |
|  | (Intervention 4. Save Last) x (Post-Intervention Month 3) |  |  |  | -0.040 | 0.373 |
|  |  |  |  |  | (0.027) |  |
|  | (Intervention 5. Save First) x (Post-Intervention Month 3) |  |  |  | -0.003 | 0.979 |
|  |  |  |  |  | (0.027) |  |
|  | (Intervention 6. Deposit-Triggered Implicit) x (Post-Intervention Month 3) |  |  |  | -0.019 | 0.688 |
|  |  |  |  |  | (0.027) |  |
|  | (Intervention 7. Deposit-Triggered Explicit) x (Post-Intervention Month 3) |  |  |  | 0.038 | 0.373 |
|  |  |  |  |  | (0.027) |  |
|  | Observations | 32,738,345 | |  | 32,738,345 | |
|  | Customer Fixed Effects | 1,925,785 | |  | 1,925,785 | |
|  | Month Fixed Effects | 17 | |  | 17 | |
|  | R-Squared | 0.949 | |  | 0.949 | |

*Note:* This table reports the results of two ordinary least squares (OLS) regressions predicting whether a given customer made any one-time transfers into their savings account at the bank in a given month. Both models include 17 months of observations for each customer (from March of 2021 - July of 2022), and both models include customer fixed effects as well as month fixed effects. In Model 1, the primary predictor variables are four interactions between an indicator for whether a customer was assigned to receive any of our megastudy’s seven intervention conditions and four indicators for the study’s two month intervention period and each of the three post-intervention months. In Model 2, the primary predictor variables are twenty-eight interactions between separate indicators for whether a customer was assigned to each of our megastudy’s seven intervention conditions and four indicators for the study’s two month intervention period and each of the three post-intervention months.Regression coefficients and standard errors have been multiplied by 100 to improve interpretability. Standard errors reported in parentheses are clustered at the customer level, and p-values in Model 2 are adjusted for multiple comparisons using the Benjamini-Hochberg procedure. * p < 0.05 ** p < 0.01 *** p < 0.001.

## Table S7. Regression-estimated impact of our megastudy’s seven intervention conditions on the change in a customer’s total savings balances during a given month of our two-month intervention or the three months following, (A) winsorizing this outcome at the 1st and 99th percentiles (Models 1-2), at the 5th and 95th percentiles (Models 3-4), at the 10th and 90th percentiles (Models 5-6) or (B) reshaping this outcome using an inverse hyperbolic sine transformation; either pooling all intervention conditions (Models 1, 3, 5 and 7), or by intervention condition (Models 2, 4, 6 and 8).

|  |  | DV: Δ in Monthly Savings  Winsorized at the 1st and 99th Pctls | | | | |  | DV: Δ in Monthly Savings  Winsorized at the 5th and 95th Pctls | | | | |  | DV: Δ in Monthly Savings  Winsorized at the 10th and 90th Pctls | | | | |  | DV: IHS(Δ in Monthly Savings) | | | | |
| --- | --- | --- | --- | --- | --- | --- | --- | --- | --- | --- | --- | --- | --- | --- | --- | --- | --- | --- | --- | --- | --- | --- | --- | --- |
|  |  | Model 1 | |  | Model 2 | |  | Model 3 | |  | Model 4 | |  | Model 5 | |  | Model 6 | |  | Model 7 | |  | Model 8 | |
| Time Period |  | *β* | *P-value* |  | *β* | *BH-Adjusted P-value* |  | *β* | *P-value* |  | *β* | *BH-Adjusted P-value* |  | *β* | *P-value* |  | *β* | *BH-Adjusted P-value* |  | *β* | *P-value* |  | *β* | *BH-Adjusted P-value* |
| During Intervention | (Assigned Any Intervention) x (Intervention-Period) | 1.601 | 0.444 |  |  |  |  | 1.815 | 0.084 |  |  |  |  | 1.183* | 0.045 |  |  |  |  | 0.014 | 0.114 |  |  |  |
|  |  | (2.092) |  |  |  |  |  | (1.051) |  |  |  |  |  | (0.591) |  |  |  |  |  | (0.009) |  |  |  |  |
|  | (Intervention 1. Savings Reminders) x (Intervention-Period) |  |  |  | 3.719 | 0.709 |  |  |  |  | 3.450 | 0.093 |  |  |  |  | 2.235* | 0.030 |  |  |  |  | 0.034* | 0.021 |
|  |  |  |  |  | (2.775) |  |  |  |  |  | (1.393) |  |  |  |  |  | (0.783) |  |  |  |  |  | (0.011) |  |
|  | (Intervention 2. Savings Wisdom Boxes) x (Intervention-Period) |  |  |  | 1.516 | 0.894 |  |  |  |  | 1.451 | 0.402 |  |  |  |  | 0.990 | 0.342 |  |  |  |  | 0.013 | 0.420 |
|  |  |  |  |  | (2.760) |  |  |  |  |  | (1.390) |  |  |  |  |  | (0.782) |  |  |  |  |  | (0.011) |  |
|  | (Intervention 3. Random Reminders) x (Intervention-Period) |  |  |  | 3.522 | 0.709 |  |  |  |  | 2.673 | 0.190 |  |  |  |  | 1.445 | 0.151 |  |  |  |  | 0.016 | 0.420 |
|  |  |  |  |  | (2.764) |  |  |  |  |  | (1.389) |  |  |  |  |  | (0.782) |  |  |  |  |  | (0.011) |  |
|  | (Intervention 4. Save Last) x (Intervention-Period) |  |  |  | -0.268 | 0.983 |  |  |  |  | 0.372 | 0.789 |  |  |  |  | 0.421 | 0.591 |  |  |  |  | 2.699E−4 | 0.981 |
|  |  |  |  |  | (2.766) |  |  |  |  |  | (1.390) |  |  |  |  |  | (0.783) |  |  |  |  |  | (0.011) |  |
|  | (Intervention 5. Save First) x (Intervention-Period) |  |  |  | 0.060 | 0.983 |  |  |  |  | 2.064 | 0.320 |  |  |  |  | 1.614 | 0.136 |  |  |  |  | 0.015 | 0.420 |
|  |  |  |  |  | (2.765) |  |  |  |  |  | (1.388) |  |  |  |  |  | (0.781) |  |  |  |  |  | (0.011) |  |
|  | (Intervention 6. Deposit-Triggered Implicit) x (Intervention-Period) |  |  |  | 1.358 | 0.894 |  |  |  |  | 1.312 | 0.402 |  |  |  |  | 0.663 | 0.462 |  |  |  |  | 0.007 | 0.596 |
|  |  |  |  |  | (2.769) |  |  |  |  |  | (1.388) |  |  |  |  |  | (0.781) |  |  |  |  |  | (0.011) |  |
|  | (Intervention 7. Deposit-Triggered Explicit) x (Intervention-Period) |  |  |  | 1.299 | 0.894 |  |  |  |  | 1.380 | 0.402 |  |  |  |  | 0.911 | 0.342 |  |  |  |  | 0.008 | 0.596 |
|  |  |  |  |  | (2.769) |  |  |  |  |  | (1.391) |  |  |  |  |  | (0.783) |  |  |  |  |  | (0.011) |  |
| 1 Month Post-Intervention | (Assigned Any Intervention) x (Post-Intervention Month 1) | -3.660 | 0.179 |  |  |  |  | -1.316 | 0.342 |  |  |  |  | -0.598 | 0.448 |  |  |  |  | -0.004 | 0.723 |  |  |  |
|  |  | (2.721) |  |  |  |  |  | (1.386) |  |  |  |  |  | (0.789) |  |  |  |  |  | (0.011) |  |  |  |  |
|  | (Intervention 1. Savings Reminders) x (Post-Intervention Month 1) |  |  |  | -4.172 | 0.573 |  |  |  |  | -0.713 | 0.814 |  |  |  |  | -0.220 | 0.833 |  |  |  |  | 0.003 | 0.951 |
|  |  |  |  |  | (3.595) |  |  |  |  |  | (1.834) |  |  |  |  |  | (1.045) |  |  |  |  |  | (0.015) |  |
|  | (Intervention 2. Savings Wisdom Boxes) x (Post-Intervention Month 1) |  |  |  | -2.355 | 0.599 |  |  |  |  | -1.364 | 0.685 |  |  |  |  | -0.938 | 0.667 |  |  |  |  | -0.010 | 0.851 |
|  |  |  |  |  | (3.602) |  |  |  |  |  | (1.834) |  |  |  |  |  | (1.045) |  |  |  |  |  | (0.015) |  |
|  | (Intervention 3. Random Reminders) x (Post-Intervention Month 1) |  |  |  | -2.768 | 0.599 |  |  |  |  | -1.518 | 0.685 |  |  |  |  | -0.934 | 0.667 |  |  |  |  | -0.014 | 0.826 |
|  |  |  |  |  | (3.597) |  |  |  |  |  | (1.833) |  |  |  |  |  | (1.045) |  |  |  |  |  | (0.015) |  |
|  | (Intervention 4. Save Last) x (Post-Intervention Month 1) |  |  |  | -3.474 | 0.586 |  |  |  |  | 0.017 | 0.993 |  |  |  |  | 0.518 | 0.723 |  |  |  |  | 0.018 | 0.826 |
|  |  |  |  |  | (3.601) |  |  |  |  |  | (1.833) |  |  |  |  |  | (1.044) |  |  |  |  |  | (0.015) |  |
|  | (Intervention 5. Save First) x (Post-Intervention Month 1) |  |  |  | -6.895 | 0.384 |  |  |  |  | -2.442 | 0.685 |  |  |  |  | -1.014 | 0.667 |  |  |  |  | -0.017 | 0.826 |
|  |  |  |  |  | (3.591) |  |  |  |  |  | (1.831) |  |  |  |  |  | (1.044) |  |  |  |  |  | (0.015) |  |
|  | (Intervention 6. Deposit-Triggered Implicit) x (Post-Intervention Month 1) |  |  |  | -1.723 | 0.631 |  |  |  |  | -1.268 | 0.685 |  |  |  |  | -0.743 | 0.667 |  |  |  |  | -0.001 | 0.951 |
|  |  |  |  |  | (3.592) |  |  |  |  |  | (1.832) |  |  |  |  |  | (1.044) |  |  |  |  |  | (0.015) |  |
|  | (Intervention 7. Deposit-Triggered Explicit) x (Post-Intervention Month 1) |  |  |  | -4.233 | 0.573 |  |  |  |  | -1.928 | 0.685 |  |  |  |  | -0.858 | 0.667 |  |  |  |  | -0.008 | 0.851 |
|  |  |  |  |  | (3.598) |  |  |  |  |  | (1.835) |  |  |  |  |  | (1.045) |  |  |  |  |  | (0.015) |  |
| 2 Months Post-Intervention | (Assigned Any Intervention) x (Post-Intervention Month 2) | 1.182 | 0.649 |  |  |  |  | 0.515 | 0.699 |  |  |  |  | 0.045 | 0.953 |  |  |  |  | 0.001 | 0.944 |  |  |  |
|  |  | (2.593) |  |  |  |  |  | (1.331) |  |  |  |  |  | (0.767) |  |  |  |  |  | (0.011) |  |  |  |  |
|  | (Intervention 1. Savings Reminders) x (Post-Intervention Month 2) |  |  |  | -0.346 | 0.920 |  |  |  |  | -0.543 | 0.985 |  |  |  |  | -0.419 | 0.843 |  |  |  |  | -0.010 | 0.950 |
|  |  |  |  |  | (3.429) |  |  |  |  |  | (1.762) |  |  |  |  |  | (1.015) |  |  |  |  |  | (0.015) |  |
|  | (Intervention 2. Savings Wisdom Boxes) x (Post-Intervention Month 2) |  |  |  | 3.825 | 0.737 |  |  |  |  | 2.127 | 0.797 |  |  |  |  | 0.949 | 0.816 |  |  |  |  | 0.006 | 0.950 |
|  |  |  |  |  | (3.424) |  |  |  |  |  | (1.763) |  |  |  |  |  | (1.015) |  |  |  |  |  | (0.015) |  |
|  | (Intervention 3. Random Reminders) x (Post-Intervention Month 2) |  |  |  | 1.649 | 0.737 |  |  |  |  | 0.034 | 0.985 |  |  |  |  | -0.403 | 0.843 |  |  |  |  | -0.003 | 0.950 |
|  |  |  |  |  | (3.440) |  |  |  |  |  | (1.764) |  |  |  |  |  | (1.015) |  |  |  |  |  | (0.015) |  |
|  | (Intervention 4. Save Last) x (Post-Intervention Month 2) |  |  |  | -3.797 | 0.737 |  |  |  |  | -1.434 | 0.971 |  |  |  |  | -1.088 | 0.816 |  |  |  |  | -0.008 | 0.950 |
|  |  |  |  |  | (3.426) |  |  |  |  |  | (1.764) |  |  |  |  |  | (1.016) |  |  |  |  |  | (0.015) |  |
|  | (Intervention 5. Save First) x (Post-Intervention Month 2) |  |  |  | 1.889 | 0.737 |  |  |  |  | 0.943 | 0.985 |  |  |  |  | 0.220 | 0.843 |  |  |  |  | 0.005 | 0.950 |
|  |  |  |  |  | (3.431) |  |  |  |  |  | (1.764) |  |  |  |  |  | (1.015) |  |  |  |  |  | (0.015) |  |
|  | (Intervention 6. Deposit-Triggered Implicit) x (Post-Intervention Month 2) |  |  |  | 1.861 | 0.737 |  |  |  |  | 0.276 | 0.985 |  |  |  |  | -0.201 | 0.843 |  |  |  |  | 0.001 | 0.950 |
|  |  |  |  |  | (3.427) |  |  |  |  |  | (1.762) |  |  |  |  |  | (1.015) |  |  |  |  |  | (0.015) |  |
|  | (Intervention 7. Deposit-Triggered Explicit) x (Post-Intervention Month 2) |  |  |  | 3.191 | 0.737 |  |  |  |  | 2.203 | 0.797 |  |  |  |  | 1.259 | 0.816 |  |  |  |  | 0.015 | 0.950 |
|  |  |  |  |  | (3.432) |  |  |  |  |  | (1.763) |  |  |  |  |  | (1.014) |  |  |  |  |  | (0.015) |  |
| 3 Months Post-Intervention | (Assigned Any Intervention) x (Post-Intervention Month 3) | 0.613 | 0.808 |  |  |  |  | -0.450 | 0.731 |  |  |  |  | -0.778 | 0.303 |  |  |  |  | -0.013 | 0.263 |  |  |  |
|  |  | (2.519) |  |  |  |  |  | (1.307) |  |  |  |  |  | (0.756) |  |  |  |  |  | (0.011) |  |  |  |  |
|  | (Intervention 1. Savings Reminders) x (Post-Intervention Month 3) |  |  |  | 3.925 | 0.686 |  |  |  |  | 1.745 | 0.959 |  |  |  |  | 0.624 | 0.746 |  |  |  |  | 0.004 | 0.770 |
|  |  |  |  |  | (3.337) |  |  |  |  |  | (1.733) |  |  |  |  |  | (1.001) |  |  |  |  |  | (0.015) |  |
|  | (Intervention 2. Savings Wisdom Boxes) x (Post-Intervention Month 3) |  |  |  | 4.068 | 0.686 |  |  |  |  | -0.058 | 0.973 |  |  |  |  | -0.659 | 0.746 |  |  |  |  | -0.012 | 0.695 |
|  |  |  |  |  | (3.342) |  |  |  |  |  | (1.734) |  |  |  |  |  | (1.002) |  |  |  |  |  | (0.015) |  |
|  | (Intervention 3. Random Reminders) x (Post-Intervention Month 3) |  |  |  | -0.348 | 0.962 |  |  |  |  | -1.028 | 0.959 |  |  |  |  | -1.210 | 0.527 |  |  |  |  | -0.009 | 0.695 |
|  |  |  |  |  | (3.324) |  |  |  |  |  | (1.727) |  |  |  |  |  | (0.999) |  |  |  |  |  | (0.015) |  |
|  | (Intervention 4. Save Last) x (Post-Intervention Month 3) |  |  |  | -1.450 | 0.935 |  |  |  |  | -1.317 | 0.959 |  |  |  |  | -1.318 | 0.527 |  |  |  |  | -0.026 | 0.267 |
|  |  |  |  |  | (3.331) |  |  |  |  |  | (1.729) |  |  |  |  |  | (1.000) |  |  |  |  |  | (0.015) |  |
|  | (Intervention 5. Save First) x (Post-Intervention Month 3) |  |  |  | 1.431 | 0.935 |  |  |  |  | 0.611 | 0.959 |  |  |  |  | -0.284 | 0.777 |  |  |  |  | -0.008 | 0.695 |
|  |  |  |  |  | (3.334) |  |  |  |  |  | (1.732) |  |  |  |  |  | (1.002) |  |  |  |  |  | (0.015) |  |
|  | (Intervention 6. Deposit-Triggered Implicit) x (Post-Intervention Month 3) |  |  |  | -3.498 | 0.686 |  |  |  |  | -2.717 | 0.813 |  |  |  |  | -2.213 | 0.188 |  |  |  |  | -0.029 | 0.267 |
|  |  |  |  |  | (3.332) |  |  |  |  |  | (1.729) |  |  |  |  |  | (1.000) |  |  |  |  |  | (0.015) |  |
|  | (Intervention 7. Deposit-Triggered Explicit) x (Post-Intervention Month 3) |  |  |  | 0.159 | 0.962 |  |  |  |  | -0.390 | 0.959 |  |  |  |  | -0.389 | 0.777 |  |  |  |  | -0.009 | 0.695 |
|  |  |  |  |  | (3.333) |  |  |  |  |  | (1.730) |  |  |  |  |  | (1.001) |  |  |  |  |  | (0.015) |  |
|  | Observations | 29,901,494 | |  | 29,901,494 | |  | 29,901,494 | |  | 29,901,494 | |  | 29,901,494 | |  | 29,901,494 | |  | 29,901,494 | |  | 29,901,494 | |
|  | Customer Fixed Effects | 1,925,785 | |  | 1,925,785 | |  | 1,925,785 | |  | 1,925,785 | |  | 1,925,785 | |  | 1,925,785 | |  | 1,925,785 | |  | 1,925,785 | |
|  | Month Fixed Effects | 16 | |  | 16 | |  | 16 | |  | 16 | |  | 16 | |  | 16 | |  | 16 | |  | 16 | |
|  | R-Squared | 0.056 | |  | 0.056 | |  | 0.089 | |  | 0.089 | |  | 0.108 | |  | 0.108 | |  | 0.132 | |  | 0.132 | |

*Note:* This table reports the results of eight ordinary least squares (OLS) regressions predicting the change in a customer’s total savings account balances during a given month after winsorization or inverse hyperbolic sine transformation. All models include 16 months of observations for each customer (from April of 2021 - July of 2022), and all models include customer fixed effects as well as month fixed effects. In Models 1, 3, 5 and 7, the primary predictor variables are four interactions between an indicator for whether a customer was assigned to receive any of our megastudy’s seven intervention conditions and four indicators for the study’s two month intervention period and each of the three post-intervention months. In Models 2, 4, 6 and 8,the primary predictor variables are twenty-eight interactions between separate indicators for whether a customer was assigned to each of our megastudy’s seven intervention conditions and four indicators for the study’s two month intervention period and each of the three post-intervention months. Standard errors reported in parentheses are clustered at the customer level, and p-values in Models 2, 4, 6 and 8 are adjusted for multiple comparisons using the Benjamini-Hochberg procedure. * p < 0.05 ** p < 0.01 *** p < 0.001.

##

## Table S8. Means and standard deviations during a given month of our two-month intervention of customers’ (1) change in total monthly savings balances and (2) monthly transfers into savings among those in the business-as-usual control condition.

|  |  |  |  |  |  |  |  |  |  |  |  |  |  |  |  |
| --- | --- | --- | --- | --- | --- | --- | --- | --- | --- | --- | --- | --- | --- | --- | --- |
|  | DV: Δ in Monthly Savings | | | | | | | | |  | DV: Monthly Transfers into Savings | | | | |
|  | Not transformed |  | Winsorized at the 1st and 99th Pctls |  | Winsorized at the 5th and 95th Pctls |  | Winsorized at the 10th and 90th Pctls |  | IHS(Δ in Monthly Savings) |  | Total Number of One-time Transfers to Savings |  | Average Size of Transfer to Savings |  | Maximum Transfer to Savings |
| Business-as-usual Control Condition | $208.36 |  | $75.97 |  | $20.41 |  | - $4.95 |  | 0.43 |  | 0.191 |  | $15.15 |  | $16.19 |
|  | (6,029.60) |  | (1,318.24) |  | (658.18) |  | (371.06) |  | (5.37) |  | (0.826) |  | (265.75) |  | (280.22) |
| Observations | 481,414 |  | 481,414 |  | 481,414 |  | 481,414 |  | 481,414 |  | 481,414 |  | 481,414 |  | 481,414 |

## Table S9. Means and standard deviations during the three months post-intervention of customers’ (1) change in total monthly savings balances and (2) whether a transfer into savings was made among those in the business-as-usual control condition.

|  |  |  |  |  |  |  |  |  |  |  |  |  |  |
| --- | --- | --- | --- | --- | --- | --- | --- | --- | --- | --- | --- | --- | --- |
|  | DV: Δ in Monthly Savings | | | | | | | | |  | DV: Whether a Transfer into Savings was Made | | |
|  | Not transformed |  | Winsorized at the 1st and 99th Pctls |  | Winsorized at the 5th and 95th Pctls |  | Winsorized at the 10th and 90th Pctls |  | IHS(Δ in Monthly Savings) |  | One-time Transfers |  | Recurring Transfers |
| Business-as-usual Control Condition 1 Month After Treatment | $61.90  (8,007.96) |  | -$53.82  (1,212.10) |  | -$59.26  (620.34) |  | -$53.43  (354.88) |  | -0.307  (5.237) |  | 8.88% |  | 13.06% |
| Business-as-usual Control Condition 2 Months After Treatment | -$0.65  (6,744.24) |  | -$63.39  (1,149.23) |  | -$59.21  (591.73) |  | -$50.41  (342.61) |  | -0.191  (5.127) |  | 9.61% |  | 15.22% |
| Business-as-usual Control Condition 3 Months After Treatment | $2.78  (5,503.27) |  | -$47.26  (1,116.16) |  | -$45.23  (578.50) |  | -$40.84  (335.26) |  | -0.083  (5.053) |  | 9.96% |  | 15.10% |
| Observations | 240,707 |  | 240,707 |  | 240,707 |  | 240,707 |  | 240,707 |  | 240,707 |  | 240,707 |

*Note:* Means for one-time transfers and recurring transfers have been multiplied by 100 to improve interpretability.

## Table S10. Clicks [on links in our emails by megastudy condition.](#_aetuxwjnd0jw)

|  | % of Customers Who Clicked Any Link(s) in Any Intervention Email | % of Customers Who Clicked A Link to “Make a One-Time Transfer” in Any Intervention Email | % of Customers Who Clicked A Link to “Make a Recurring Transfer” in Any Intervention Email |
| --- | --- | --- | --- |
| Intervention 1. Savings Reminders | 2.58% | 0.39% | 1.69% |
|  |  |  |  |
| Intervention 2. Savings Wisdom Boxes | 2.70% | 0.45% | 1.75% |
|  |  |  |  |
| Intervention 3. Random Reminders | 1.01% | 0.10% | 0.74% |
|  |  |  |  |
| Intervention 4. Save Last | 0.87% | 0.11% | 0.58% |
| Intervention 5. Save First | 0.93% | 0.11% | 0.65% |
|  |  |  |  |
| Intervention 6. Deposit-Triggered Implicit | 0.62% | 0.06% | 0.47% |
|  |  |  |  |
| Intervention 7. Deposit-Triggered Explicit | 1.10% | 0.15% | 0.75% |

## Table S11. Summary statistics for the full sample and each condition as well as a balance test comparing the total number of non-intervention emails sent to customers, on average, by our banking partner during our study’s two-month intervention period (March and April of 2022) across conditions. Notably, more non-intervention emails were sent in the control condition than the treatment conditions given bank rules regarding daily email limits (see main manuscript for discussion).

|  | Average Number of Non-Intervention Emails Sent by the Bank to Each Customer (Std. Dev.) |
| --- | --- |
|  |  |
| Total Sample | 11.12 |
|  | (4.55) |
| Control Condition | 11.24 |
|  | (4.55) |
| Intervention 1. Savings Reminders | 11.06 |
|  | (4.60) |
| Intervention 2. Savings Wisdom Boxes | 11.07 |
|  | (4.60) |
| Intervention 3. Random Reminders | 11.10 |
|  | (4.53) |
| Intervention 4. Save Last | 11.10 |
|  | (4.53) |
| Intervention 5. Save First | 11.19 |
|  | (4.58) |
| Intervention 6. Deposit-Triggered Implicit | 11.10 |
|  | (4.50) |
| Intervention 7. Deposit-Triggered Explicit | 11.11 |
|  | (4.50) |
| F-statistic for F-test of equality across conditions | 44.306 |
|  | P < 0.001 |

*Note*: This table reports the mean number of non-intervention emails sent to customers by our banking partner during our study’s two-month intervention period (March and April of 2022) for our full customer sample and for the subset of customers in each study condition (standard deviations in parenthesis). We also report the results of an F-test for equality across conditions and find imbalance, which is not surprising given that the bank limited the total number of emails a customer could receive per day. This rule meant customers in our control condition (and in conditions that triggered fewer messages) had more bandwidth to receive non-intervention emails than those in our treatment conditions.
